# A quantitative sequencing method using synthetic internal standards including functional and phylogenetic marker genes

**DOI:** 10.1111/1758-2229.13189

**Published:** 2023-07-18

**Authors:** Kazuyoshi Koike, Ryo Honda, Masataka Aoki, Ryoko Yamamoto‐Ikemoto, Kazuaki Syutsubo, Norihisa Matsuura

**Affiliations:** ^1^ Graduate School of Natural Science and Technology Kanazawa University Kanazawa Japan; ^2^ Faculty of Geosciences and Civil Engineering Kanazawa University Kanazawa Japan; ^3^ Regional Environment Conservation Division National Institute for Environmental Studies (NIES) Ibaraki Japan; ^4^ Research Center for Water Environment Technology, School of Engineering the University of Tokyo Tokyo Japan

## Abstract

The method of spiking synthetic internal standard genes (ISGs) to samples for amplicon sequencing, generating sequences and converting absolute gene numbers from read counts has been used only for phylogenetic markers and has not been applied to functional markers. In this study, we developed ISGs, including gene sequences of the 16S rRNA, *pmoA*, encoding a subunit of particulate methane monooxygenase and *amoA*, encoding a subunit of ammonia monooxygenase. We added ISGs to the samples, amplified the target genes and performed amplicon sequencing. For the mock community, the copy numbers converted from read counts using ISGs were equivalent to those obtained by the quantitative real‐time polymerase chain reaction (4.0 × 10^4^ versus 4.1 × 10^4^ and 3.0 × 10^3^ versus 4.0 × 10^3^ copies μL‐DNA^−1^ for 16S rRNA and *pmoA* genes, respectively), but we also identified underestimation, possibly due to primer coverage (7.8 × 10^2^ versus 3.7 × 10^3^ μL‐DNA^−1^ for *amoA* gene). We then applied this method to environmental samples and analysed phylogeny, functional diversity and absolute quantities. One *Methylocystis* population was most abundant in the sludge samples [16S rRNA gene (3.8 × 10^9^ copies g^−1^) and the *pmoA* gene (2.3 × 10^9^ copies g^−1^)] and were potentially interrelated. This study demonstrates that ISG spiking is useful for evaluating sequencing data processing and quantifying functional markers.

## Graphical abstract

We designed four synthetic internal standard genes containing sequences of phylogenetic and functional marker genes. These synthetic ISGs are spike‐in to the sample DNA and PCR amplification and amplicon sequencing are performed to determine both the sequence and quantity of the gene. This method will promote the study of microbial dynamics in natural and engineered systems by simplifying the design of research methods that combine phylogenetic, functional diversity and quantitative analysis of microorganisms.

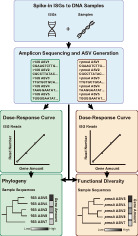

## INTRODUCTION

Microbial communities are intricately linked to biochemical processes in both natural and engineered systems. Various molecular biological approaches have been developed to understand the response of microbial ecosystems to environmental changes and the association between their diversity and processes (Martiny et al., 2015). High‐throughput 16S ribosomal RNA (16S rRNA) gene amplicon sequencing is an indispensable method for analysing the composition and dynamics of microorganisms. In natural systems, such as soil (Pérez Castro et al., 2019; Hao et al., 2021) and water (Morrison et al., 2017; Teachey et al., 2019), 16S rRNA gene amplicon sequencing analysis is used to confirm the effects of environmental factors on the composition and diversity of microorganisms. Microbial analysis is also used to assess and monitor the composition of microbial communities in artificial ecosystems, such as methane fermentation (Shimizu et al., 2022; Yu et al., 2018) and biological water treatment processes (Liang et al., 2020; Matsuura et al., 2021).

However, the dynamics observed using that approach are based on relative abundance and may not accurately reflect the actual dynamics of taxonomic density. It has been reported that increases in relative abundance are not necessarily associated with increases in absolute abundance (Props et al., 2017). Therefore, measuring absolute amounts is important to understand microbial dynamics. The robust microbial quantification methods include direct cell counting using flow cytometry (Hammes et al., 2008) and fluorescence in situ hybridization (Amann & Fuchs, 2008). Moreover, quantitative real‐time polymerase chain reaction (qPCR) is a widely used method in microbial quantification, which can measure gene levels more rapidly and with higher sensitivity and can indirectly quantify cell numbers (Geets et al., 2007). Therefore, by combining the total gene abundance obtained from qPCR with the relative proportion of individual taxonomy obtained from amplicon sequencing, the gene abundance of individual taxonomy can be estimated (Dannemiller et al., 2014). This method is widely used to elucidate microbial dynamics (Harms et al., 2003) but is limited by the time required due to different workflows for quantitation and sequencing and high initial costs owing to the need for separate equipment.

In recent years, quantitative sequencing methods have been developed to spike microbial DNA (Lin et al., 2018; Smets et al., 2016) or viable bacteria absent in the sample (Stämmler et al., 2016), or to add synthetic 16S rRNA (Tourlousse et al., 2017) or 16S‐18S‐internal transcribed spacer regions (Tkacz et al., 2018; Wang et al., 2021) as standards for high‐throughput quantitative microbial community analysis. These quantitative sequencing methods can be divided into two quantitative patterns: (i) the total number of a target gene in a sample is calculated from the internal standard gene (ISG) read ratio and the total number of ISG molecules added to the sample (Galagoda et al., 2023; Lin et al., 2018; Smets et al., 2016; Stämmler et al., 2016; Tkacz et al., 2018) or (ii) the target gene read numbers are converted to copy numbers using a dose–response curve as an internal standard curve, which is estimated based on the ISG read numbers with known gene copy numbers (Tourlousse et al., 2017; Wang et al., 2021). However, neither method is widespread enough to replace qPCR.

These high‐throughput amplicon 16S rRNA gene quantification methods are powerful tools for elucidating microbial dynamics, while the molecular phylogeny of 16S rRNA genes does not allow the direct identification of the metabolic functions of each microorganism. Quantitative DNA stable isotope probing (DNA‐qSIP) is a powerful method for measuring microbial metabolism and activity but requires culture and fractionation of stable isotope‐labelled and unlabelled DNA (Coskun et al., 2018; Vuillemin et al., 2019). Additionally, diversity analyses targeting functional marker genes encoding enzymes provide us with information on potential metabolic functions without culture and complex operations (Knief, 2015; Müller et al., 2015; Pester et al., 2014; Purkhold et al., 2003). Thus, high‐throughput quantitative methods focused on functional markers are powerful tools for elucidating biochemical processes and microbial dynamics in both natural and engineered systems.

Although metagenomic sequencing and downstream analysis tools are easy to use these days, PCR‐based targeted gene quantification and sequencing remains a powerful tool. This is because the metagenomic‐based phylogenetic and functional analysis approaches have limitations including insufficient sample amount and the possibility of missing rare microbes. Thus, the goal of this study is to develop a sequencing‐based, functional gene quantification method comparable to the frequently used qPCR method. In this study, we established a high‐throughput absolute quantification method for functional genes using internal standard calibration to detect and quantify microorganisms with specific metabolic functions from complex microbial communities in the environment. Methane‐oxidizing (methanotrophs) and ammonia‐oxidizing bacteria (AOB), and their well‐studied functional genes, *pmoA* (encoding particulate methane monooxygenase) and *amoA* (encoding ammonia monooxygenase), were analysed. Synthetic ISG sequences were designed with two primer sequences targeting the *pmoA* gene, one targeting the *amoA* gene, two targeting the 16S rRNA gene, and spacer sequences to fill in the gaps between each primer sequence. Four variable ISGs were created by changing the spacer sequences. We evaluated this new method using a mock community and then applied it to environmental samples.

## EXPERIMENTAL PROCEDURES

### Design and synthesis of ISG sequences

**FIGURE 1:**
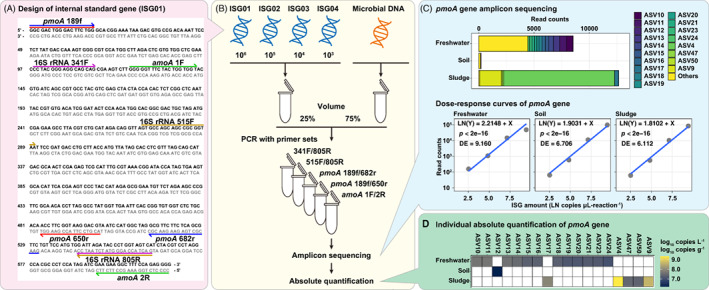
Workflow from the design of synthetic internal standard genes (ISGs) to microbial gene quantification. (A) Design of synthetic ISGs and location of incorporated primer sets. (B) Schematic diagram from amplicon sequencing library preparation to absolute quantitation. (C) Individual amplicon sequence variant (ASV) estimation and acquisition of dose–response curves from amplicon sequencing data. (D) Absolute quantification of individual ASVs using internal standard curves.

The workflow of this quantitative sequencing method using synthetic internal standards is shown in Figure 1. First, we designed a set of synthetic ISG sequences consisting of primer sequences targeting the *pmoA* gene (*pmoA* 189f/682r and 650r), *amoA* gene (*amoA* 1F/2R) and 16S rRNA gene (341F and 515F/805R) (Figure 1A, Table 1). Spacers between primers were filled using artificially designed 16S rRNA gene variable region sequences (Ec5001, Ec5002, Ec5003 and Ec5004) (Tourlousse et al., 2017) to adjust the amplification length and avoid primers being adjacent to each other. The primer sequences incorporated into the ISGs were arranged as amplified fragment lengths of 500, 531, 491, 465 and 291 bp for *pmoA* 189f/650r, *pmoA* 189f/682r, *amoA* 1F/2R, 341F/805R and 515F/805R primer sets, respectively. To ensure that the primers used for amplicon sequencing did not match the gap sequence, alignment was performed using ClustalW v2.1 (Thompson et al., 1994). If a match was expected, the gap sequence was replaced with other bases. The overall guanine‐cytosine (GC) content was maintained at approximately 50% by replacing some of the G and C bases in the gap with adenine (A) and tyrosine (T) bases, and the GC content of the synthetic ISGs varied between 51.5% and 52.8% (Table S3). BLASTN was performed for the four designed ISGs (Table S3) using the National Center for Biotechnology Information (NCBI) nucleotide collection (nr/nt) database and they were found to be non‐identical to any known sequence, except for synthetic spike‐in controls (Tourlousse et al., 2017) (web‐BLASTN performed in October 2018).

**TABLE 1:** Primer lists and thermocycling protocols.

Gene target	Primer name	Primer sequence (5′‐3′)	Product length (bp)	Reference
*pmoA* ^a^	*pmoA* 189f	GGNGACTGGGACTTCTGG	500	Holmes et al., 1995
	*pmoA* 650r	ACGTCCTTACCGAAGGT		Bourne et al., 2001
*pmoA* ^b^	*pmoA* 189f	GGNGACTGGGACTTCTGG	531	Holmes et al., 1995
	*pmoA* 682r	GAASGCNGAGAAGAASGC		
Nitrosomonadaceae AOB *amoA* ^c^	*amoA* 1F	GGGGTTTCTACTGGTGGT	491	Rotthauwe et al., 1997
	*amoA* 2R	CCCCTCKGSAAAGCCTTCTTC		
16S rRNA V3‐4 region^d^	341F	CCTACGGGNGGCWGCAG	465	Herlemann et al., 2011
	805R	GACTACHVGGGTATCTAATCC		
16S rRNA V4 region^e^	515F	GTGCCAGCMGCCGCGGTAA	291	Caporaso et al., 2012
	805R	GACTACHVGGGTATCTAATCC		Herlemann et al., 2011

*Note*: Illumina adapter sequence: Forward sequence, 5′‐TCGTCGGCAGCGTCAGATGTGTATAAGAGAAG‐3′; Reverse sequence, 5′‐GTCTCGTGGGCTCGGAGATGTGTATAAGAGACAG‐3′. Thermocycling protocol: ^a^Initial heat activation, 95°C 15 min; Amplification for 30 cycles, 94°C 30 s, 50°C 45 s, 72°C 1 min; Final extension, 72°C 10 min. ^b^Initial heat activation, 95°C 15 min; Amplification for 30 cycles, 94°C 30 s, 56°C 1 min, 72°C 1 min; Final extension, 72°C 10 min. ^c^Initial heat activation, 95°C 15 min; Amplification for 30 cycles, 94°C 30 s, 55°C 40 s, 72°C 1 min; Final extension, 72°C 10 min. ^d^Initial heat activation, 95°C 15 min; Amplification for 25 cycles, 94°C 30 s, 50°C 1 min, 72°C 1 min; Final extension, 72°C 10 min. ^e^Initial heat activation, 95°C 15 min; Amplification for 25 cycles, 94°C 30 s, 53°C 30 s, 72°C 1 min; Final extension, 72°C 10 min. For qPCR, Amplification for 40 cycles; Melt curve, 95°C 1 min, 55°C 30 s, 95°C 30 s.

### Preparation of internal standards

Full‐length ISG sequences (615 bp) were chemically synthesized and inserted into the pEX‐K4J2 vector (Eurofins Genomics, Japan). ISG sequences inserted into the plasmid vectors were amplified using specific primer sequences (primer sequences and thermocycling protocols in Table S4). Initially, plasmid vectors were diluted with Tris‐EDTA (TE) buffer (pH 8.0) (NIPPON GENE, Japan) in 1.0 × 10^5^ copies μL^−1^ to use as the PCR template. PCR amplification was performed in 50 μL reactions containing 1× PCR buffer, 200 nM each of the forward and reverse primers (Eurofins Genomics, Japan), 200 μM of each deoxynucleotide triphosphate, 0.5 units of HotStarTaq DNA Polymerase (Qiagen, Germany) and 2.0 μL of template. Amplified ISGs were purified using the Agencourt AMPure XP system with 1.0 volumes of AMPure beads (Beckman Coulter, USA) and eluted in 45 μL of UltraPure™ DNase/RNase‐Free Distilled Water (Thermo Fisher Scientific, USA). All DNA used in this study was handled using the Axygen MAXYMum Recovery product (CORNING, USA). The size and concentration of the ISGs were measured using the DNA 1000 assay system and 2100 Bioanalyzer (Agilent Technologies, USA). The purified ISG concentrations were calculated using equation (i):

$$C\left(\text{copies}\;{\mathrm{\mu L}}^{-1}\right)=\frac{M\left(\mathrm{ng}\;{\mathrm{\mu L}}^{-1}\right)\times 6.02\times 10^{23}\left({\mathrm{mol}}^{-1}\right)}{660\left(g\;{\mathrm{mol}}^{-1}\;{\mathrm{bp}}^{-1}\right)\times 10^{9}\left(\mathrm{ng}\;g^{-1}\right)\times \mathrm{DNA}\;\text{length}\left(\mathrm{bp}\;{\text{copies}}^{-1}\right)},$$

where *C* is the ISG concentration, *M* is the mass of the ISG and the DNA length is 615 bp. The purified ISGs were diluted to 4.0 × 10^8^ copies μL^−1^ in TE buffer (pH 8.0) and distributed in single‐use aliquots stored at −20°C. Gradient dilutions of the four internal standards (ISG01–ISG04) were prepared as 4.0 × 10^6^, 10^5^, 10^4^ and 10^3^ copies μL^−1^ and mixed with equal volumes to form gradient internal standard concentrations (total concentration was 1.111 × 10^6^ copies μL^−1^).

### Mock community preparation and DNA extraction

*Methylomagnum ishizawai* strain RS11D‐Pr (NBRC 109438), *Nitrosomonas europaea* ATCC 19718 (NBRC 14298) and *Escherichia coli* (NBRC 3301) were cultured according to the instructions of the National Institute of Technology and Evaluation Biological Resource Center (NBRC); *M. ishizawai* contains the *pmoA* gene encoding pMMO and *N. europaea* contains the *amoA* gene encoding AMO, but *E. coli* does not contain either of them. A mock community was then prepared using genomic DNA extracts from these microorganisms. Genomic DNA was extracted from each fully grown culture separately using phenol and purified using phenol/chloroform/isoamyl alcohol and chloroform/isoamyl alcohol. The genomic DNA was treated with deoxyribonuclease‐free ribonuclease in glycerol solution to minimize RNA contamination (see Data S1 for details), stored at 4°C and then used for qPCR and amplicon sequencing library preparation. The mass of the genomic DNA was measured using an Eppendorf BioPhotometer D30 (Eppendorf, Germany), and three types of genomic DNA concentrations were estimated according to equation (i), which were mixed at equivalent amounts (3.0 × 10^4^ copies μL^−1^ each) to create a mock community. In equation (i), *C* is the genomic DNA concentration, *M* is the mass of the genomic DNA, and the DNA (genome) lengths of *M. ishizawai*, *N. europaea* and *E. coli* were calculated to be 4.61 Mbp (GenBank accession number: AP019783), 2.81 Mbp (AL954747) and 4.75 Mbp (NZ_BJLE00000000), respectively.

### qPCR assay

The numbers of *pmoA*, *amoA* and 16S rRNA genes in the mock community were measured by qPCR assay, using primers without Illumina adapter sequences (primer sequences and thermocycling protocols in Table 1). All DNA extracts and ISGs were handled using Axygen MAXYMum Recovery products (CORNING, USA) to minimize DNA loss in microtubes and pipette tips. For the functional gene standard preparation, the genomic DNA of *M. ishizawai* and *N. europaea* were used to amplify *pmoA* and *amoA* genes, respectively, using primers with Illumina adapter sequences attached to the 5′ end of the forward and reverse primers (Table S5). The approximate full‐length 16S rRNA gene of *E. coli* was amplified using primers without Illumina adapter sequences (Table S5) for its gene standard preparation. Each standard curve was prepared from a 10‐fold serial dilution of the amplified gene (4.0 × 10^6^–10^2^ copies μL^−1^). All qPCR assays were carried out on an Agilent Technologies Stratagene Mx3000P Real‐Time qPCR system (Agilent Technologies, USA) in a total volume of 20 μL containing 1× QuantiTect SYBR Green PCR Master Mix (QIAGEN, Germany), 200 nM of forward and reverse primers each (synthesized by Eurofins Genomics, Japan) and 2.0 μL of DNA template. All reactions were performed in biological triplicate (*n* = 3). Ct values were obtained using the MxPro qPCR Software (Agilent Technologies, USA), and gene copy numbers were calculated using calibration curves. The calculated number of gene copies per unit volume of each reaction was converted to that of DNA used for qPCR using equation (ii):

$$Z_{\text{gene conc}.}=\frac{\frac{Y_{\text{gene conc}.}\times D_{\text{sample dilution}\;\mathrm{vol}.}}{E_{\text{diluted}\;\mathrm{vol}.}}\times H_{\text{qPCR}\;\mathrm{use}\;\mathrm{vol}.}}{I_{\text{qPCR reaction}\;\mathrm{vol}.}},$$

where *Z*_*gene conc*._ (copies μL‐reaction^−1^) is the qPCR gene concentration using the linear predictor, *Y*_*gene conc*._ (copies μL‐DNA^−1^) is the gene concentration in the sample after DNA extraction, *D*_*sample dilution vol*._ (μL) is the volume of DNA used for dilution, *E*_*diluted vol*._ (μL) is the volume of DNA after dilution, *H*_*qPCR use vol*._ (μL) is the volume of the sample used for qPCR and *I*_*qPCR reaction vol*._ (μL) is the qPCR reaction volume.

### Environmental sample collection and DNA extraction

Sludge sample was squeezed out of the sponge in a full‐scale biological ammonium removal reactor system used for drinking water production in Japan on 1 November 2019 (details are described in our previous report (Koike et al., 2022), equivalent to 1013 days in operation). Freshwater samples were collected from the surface of the Phra Khanong canal, in Thailand on 17 April 2018. On 11 January 2019, rice paddy soil samples were collected from a paddy field in Kanazawa City, Japan, by removing approximately 5 mm of the surface layer. These samples were stored at 4°C in 50 mL sterile centrifuge tubes and transferred to the laboratory immediately after collection. The sludge and soil samples were washed once with 1× phosphate‐buffered saline and pelleted (10,000*g*, 5 min, 4°C) before DNA extraction. DNA was extracted using the FastDNA SPIN Kit for Soil (MP Biomedicals, USA) from 0.2 g of the washed pellet, as described previously (Koike et al., 2022). Four millilitres of the freshwater sample was vacuum filtered using a mixed cellulose ester membrane with 0.2 μm pore size (ADVANTEC, Japan), and the membrane was subsequently used instead of the pellet for DNA extraction, which was performed as described above. The extracted DNA samples were stored at −20°C and the mass of DNA was measured using an Eppendorf BioPhotometer D30. The DNA concentrations of the environmental samples were estimated using equation (i), where *C* is the estimated genomic DNA concentration of environmental samples, *M* is the mass of the DNA and DNA (assumed genome) length is 3.85 Mbp (the average genome length of only the prokaryotes from the Genome Information by Organism of NCBI database was used; the data set was obtained in December 2018). Environmental DNA samples were diluted to 1.111 × 10^6^ copies μL^−1^ in TE buffer (pH 8.0), stored at 4°C, and then immediately used in the qPCR and amplicon sequencing library preparation.

### Amplicon sequencing library preparation and Illumina sequencing

DNA libraries for *pmoA*, *amoA* and 16S rRNA gene amplicon sequencing were prepared using a two‐step tailed PCR method and primers (primer sequences in Table 1). Illumina adapter sequences were attached to the 5′ end of the forward and reverse sequencing primers, respectively. Concentration gradient diluted internal standards were added to the mock community or environmental DNA samples at a ratio 1:3 to obtain template DNA. All PCR amplification rounds were run using a T100 Thermal Cycler (Bio‐Rad, USA). We performed 50 μL reactions for the first round of PCR containing 1× PCR buffer, 200 nM each of the forward and reverse primers (Eurofins Genomics, Japan), 200 μM of dNTP mixture (TaKaRa, Japan), 0.5 units of HotStarTaq DNA Polymerase (Qiagen, Germany) and 2.5 μL of DNA template (concentration of 2 ng μL^−1^) with the thermocycling conditions indicated in Table 1. Then, we purified the PCR amplicons using the Agencourt AMPure XP system with 1.0 volume of AMPure beads (Beckman Coulter, USA) and eluted it in 45 μL of UltraPure™ DNase/RNase‐Free Distilled Water (Thermo Fisher Scientific, USA).

Following that, we conducted 25 μL reactions for the second round of PCR containing 1× KAPA HiFi HotStart ReadyMix (Roche, Switzerland), 300 nM each of the index 1 (i7) and index 2 (i5) Nextera XT index adapters (Illumina, USA), and 2 μL of purified first round PCR products. Thermocycling was carried out under the following conditions: 95°C for 3 min; 8 cycles of 98°C for 20 s, 60°C for 15 s and 72°C for 1 min; and 72°C for 1 min. The second round PCR products were purified with 1.0 volume of AMPure XP beads, quantified using the DNA 1000 assay system and the 2100 Bioanalyzer instrument (Agilent Technologies, USA), diluted to 2 nM, aliquoted into 10 μL each and pooled to prepare libraries for multiplex sequencing. Library preparation for the mock community was performed in biological duplicates (*n* = 2). Finally, the prepared libraries were sequenced on the Illumina MiSeq platform using the MiSeq Reagent Kit v3 (600 cycle; Illumina, USA), producing 350 and 250 bp paired reads. PhiX Control v3 (Illumina) was added to the libraries at a concentration of approximately 40% (v/v).

### Amplicon sequence analysis

Illumina sequence reads were analysed using the following bioinformatics tools: Trimmomatic v0.36 (Bolger et al., 2014), Cutadapt v1.12 (Martin, 2011) and DADA2 v1.16.0 (Callahan et al., 2016). First, sequences of less than 75 bases were removed (option MINLEN:75) from the raw paired‐end (PE) data sets, and quality trimming (SLIDINGWINDOW:10:15) was performed using Trimmomatic. Second, primers were removed using Cutadapt, allowing up to two mismatches (−e 0.12) and reads without adapters were discarded (‐‐discard‐untrimmed). Third, each gene data set was filtered and trimmed (truncLen, maxEE) with better parameter values via DADA2. DADA2 was used for error rate estimation (nbases=1e10), dereplicating the reads (default parameters), amplicon sequence variant (ASV) inference (pool=TRUE), read‐pair merging and chimaera removal (default parameters) in all data sets. ASVs were taxonomically classified using our in‐house database for mock community analysis. For environmental sample analysis, the 16S rRNA gene amplicons were taxonomically classified using the suggested publicly available training data set derived from the SILVA 138 SSU Ref NR 99 database (Quast et al., 2012), while *pmoA* and *amoA* gene amplicons were classified using our in‐house database and confirmed via BLASTN to the NCBI web server. ISG sequences were added to all reference databases used in the DADA2 process. Finally, we checked the ISG and mock community sequences of the ASVs and manually removed any ASV reads that had mismatches with the reference sequence. Hereafter, reads before the DADA2 process are called read‐in whereas those after the DADA2 process are called read‐out.

The quality of Illumina sequencing reads being significantly lower in later cycles has been previously reported (Ramakodi, 2021). To minimize read trimming caused by quality descent, ASV estimation was performed on single‐end (SE) 251 bp high‐quality filtered forward data sets (truncLen=c(251), maxEE=c(2)) without merging. The read recovery rate and quantity of the mock community were then compared between the PE and SE data sets.

### Absolute quantification of mock communities and environmental samples using synthetic ISGs

Data processing and analysis were performed in the R statistical computing environment v4.0.4 (https://www.r-project.org/) (R Core Team, 2018). To determine the absolute number of microbial genes in the mock community and environmental samples, read counts were regressed to ISG amounts by a generalized linear model (GLM) with negative binomially distributed errors using the function ‘glm.nb’ of the R package MASS v7.3.53 (https://cran.r-project.org/package=MASS) (Venables & Ripley, 2002). Standard curves for absolute quantification were obtained using the ‘glm.nb’, with an offset‐fixed slope of 1. The linear predictor is expressed as equation (iii) with the natural logarithmic link function:

$$\mathrm{LN}\left(Y\right)=X+b,$$

where *Y* is the sequencing read number, *X* is the gene concentration (LN [copies μL‐reaction^−1^]) and *b* is the estimated intercept. To convert the sequencing read counts to gene concentration, it was regressed on an exponential function of the natural number. The calculated number of gene copies per unit volume of each reaction was converted to the number of gene copies per volume of DNA used for PCR, using equation (iv):

$$Z_{\text{gene conc}.}=\frac{\frac{\frac{Y_{\text{gene conc}.}\times D_{\text{sample dilution}\;\mathrm{vol}.}}{E_{\text{diluted}\;\mathrm{vol}.}}\times F_{\text{sample}\;\mathrm{use}\;\mathrm{vol}.}}{G_{\text{sample}+\mathrm{ISG}\;\mathrm{vol}.}}\times H_{\mathrm{PCR}\;\mathrm{use}\;\mathrm{vol}.}}{I_{\mathrm{PCR}\;\text{reaction}\;\mathrm{vol}.}},$$

where *Z*_*gene conc*._ (copies μL‐reaction^−1^) is the gene concentration converted from read counts using the linear predictor, *Y*_*gene conc*._ (copies μL‐DNA^−1^) is the gene concentration in the sample after DNA extraction, *D*_*sample dilution vol*._ (μL) is the volume of DNA used for dilution, *E*_*diluted vol*._ (μL) is the volume of DNA after dilution, *F*_*sample use vol*._ (μL) is the volume of post‐dilution DNA used to create the sample and ISG mixture, *G*_*sample+ISG vol*._ (μL) is the sample and ISG mixture volume, *H*_*PCR use*_ *vol*. (μL) is the volume of the sample and ISG mixture used for PCR and *I*_*PCR reaction vol*._ (μL) is the PCR reaction volume. Additionally, the number of genes per DNA volume of environmental samples was converted to the number of genes per sample weight or volume assuming 100% DNA extraction efficiency using the following equation (v):

$$X_{\text{gene conc}.}=\frac{Y_{\text{gene conc}.}\times C_{\mathrm{DNA}\;\text{extraction}\;\mathrm{vol}.}}{A_{\text{sample}\;\mathrm{wt}.\;\text{or}\;\mathrm{vol}.}},$$

where *X*_*gene conc*._ (copies g^−1^ or copies L^−1^) is the assumed gene concentration in the environmental sample, *Y*_*gene conc*._ (copies μL‐DNA^−1^) is the gene concentration in the sample after DNA extraction, *C*_*DNA extraction vol*._ (μL) is the DNA concentration of the sample and *A*_*sample wt. or vol*._ (g or L) is the sample weight or volume.

### Phylogenetic analysis

The methanotrophic 16S rRNA and *pmoA* gene sequences were collected from the complete and metagenome‐assembled genomes reconstructed from cultures or environments in the NCBI or the Integrated Microbial Genomes database provided by the Joint Genome Institute. To identify the closely related methanotroph species detected in our environmental samples, only ASVs with read counts above the lower limit of the detected concentration gradient internal standards were imported into our custom‐made 16S rRNA or *pmoA* gene database. To construct a phylogenetic tree based on the methanotrophic 16S rRNA gene, the 16S ASVs and reference sequences were aligned using MAFFT v7.215 (Katoh et al., 2002). Thereafter, the aligned sequences were used to calculate maximum likelihood (ML) trees using IQ‐TREE v2.1.3 (Minh et al., 2020), with a model determined by an automatic search of the best‐fit model (−m MFP) and 1000 replications of ultrafast bootstrapping (‐B 1000). For the diversity analysis of methanotrophs, *pmoA* ASVs and reference nucleotide sequences were translated to PmoA amino acid sequences using the FrameBot tool v1.2 (Wang et al., 2013). PmoA amino acid sequences were then aligned using MAFFT, and ML trees were calculated using IQ‐TREE with the same settings as those used for the 16S rRNA gene‐based ML tree.

## RESULTS

### Read recovery ratio of PE and SE forward read data sets in mock communities pooled with ISGs

**FIGURE 2:**
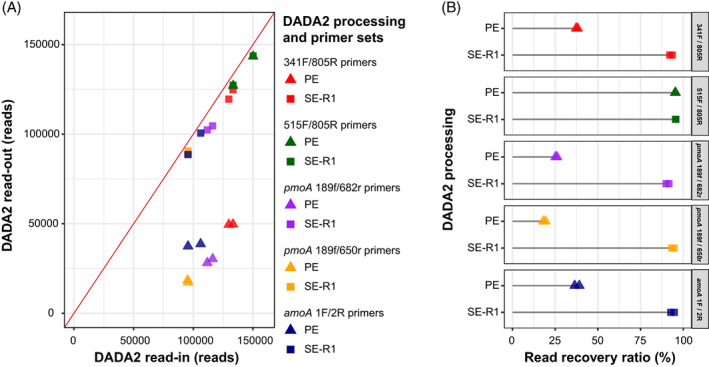
Comparison of DADA2 processing methods for amplicon sequencing data. Amplicon sequencing of each primer set was performed in duplicate and plotted in the figure, respectively. (A) Read counts before and after DADA2 processing. The paired‐end (PE) data set is shown in a triangle plot, the single‐end forward (SE‐R1; 251 bp) data set in a square plot, the differences in the data set for each primer set in colour, and 100% recovery efficiency as a red line. (B) Comparison of read recovery ratio in PE and SE‐R1 for read‐out against DADA2 read‐in. The read recovery ratio was calculated using the total number of reads after initial quality control as input and the total number of reads after the DADA2 process as output.

To evaluate the read merging process, we compared the number of reads before (read‐in) and after (read‐out) DADA2 processing. In read‐out, the number of reads decreased significantly except for the 515F/805R primer set (Figure 2A), and in PE data sets undergoing the merging process, the recovery ratio was less than 40% for all primer sets except 515F/805R (Figure 2B), and the recovery ratio appeared to decrease as the read length after merging increased (Figure S1). Therefore, we estimated the ASV using only 251 bp of high‐quality SE‐R1 data sets. Although we need to carefully compare the 16S rRNA gene phylogeny with different hyper‐variable regions or functional gene diversity with shorter length sequencing data (Fuks et al., 2018; Johnson et al., 2019), SE‐R1 was shown to have a class‐level microbial core structure comparable to that of merged PE in a recent report on environmental microbial analysis (Ramakodi, 2021). In this study, recovered read numbers were increased (Figure 2A) and more than 90% of reads persisted in all primer sets (Figure 2B). These results indicate that using the SE‐R1 data sets in this study also prevents a reduction in the sequencing depth. Interestingly, the difference in read recovery ratio for the mock community against the mock community reads plus ISG reads was 1.0‐, 0.9‐, 13.0‐, 57.2‐ and 1.1‐fold for sequencing primer sets 341F/805R, 515F/805R, *pmoA* 189f/682r, *pmoA* 189f/650r and *amoA* 1F/2R, respectively, and were more pronounced in the *pmoA* gene (Figure S2). This means that there is a difference in read recovery ratio between the mock community gene and ISG in the case of the *pmoA* gene, which is expected to lead to differences in absolute quantitative results between the PE and SE‐R1 data sets when using the standard curve to convert read counts to gene concentration.

### Absolute quantification of mock community genes

**FIGURE 3:**
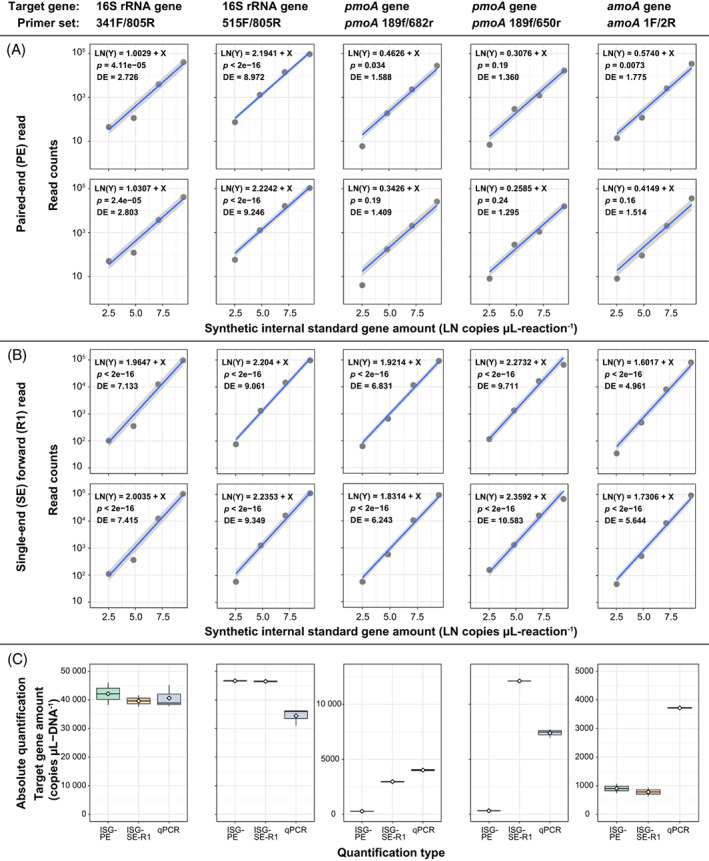
Assessment of the quantitative performance of the synthetic internal standard genes (ISGs). Dose–response curves for individual absolute quantification for the (A) paired‐end (PE) data set and (B) single‐end forward (SE‐R1; 251 bp) data set. Amplicon sequencing was performed in duplicate with each primer set. (C) Box plot of total gene copy number of the mock community with read counts converted from standard curve intercepts to absolute copy numbers. A diamond in the box plot indicates the mean value and the black horizontal line indicates the median (ISG, *n* = 2; quantitative real‐time polymerase chain reaction, *n* = 3).

We obtained an internal standard curve using the theoretical synthetic ISG amount (copies per μL reaction volume; copies μL‐reaction^−1^) and ASV read counts within the mock community. Internal standard curves for absolute quantitation were obtained by negative binomial GLM regression analysis of dose–response curves, with the slope of the model fixed at 1. The fitted intercept was assigned as the linear predictor and the read counts were converted to absolute copy numbers. For quantitative evaluation, we compared the detection efficiency (DE), expressed as the number of reads per copy (reads copy ^−1^) of mock community genes. In the PE data set, the *p*‐value from the Wald test (a value of 0.05 or less indicated that the coefficient of the explanatory variable was valid) did not show significance in the functional gene data set, and there was a divergence in DE between the high read recovery primer sets 515F/805R (DE = 9.0–9.2) and the others (DE = 1.3–2.8) (Figure 3A). When ASV was estimated using SE‐R1 (251 bp), *p*‐values were lower than 0.05 for all data sets (*p* < 2e‐16), and DE values for all primer sets varied between 5.0 and 10.6 (Figure 3B). Thus, the SE‐R1 data sets provide more significant internal standard curves and higher read DE than the PE data sets.

We compared the number of genes per extracted DNA volume of the mock community using three methods: (i) quantification using the PE data set (ISG‐PE, *n* = 2), (ii) quantification using the SE‐R1 data set (ISG‐SE‐R1, *n* = 2) and (iii) qPCR (n = 3) (Figure 3C). The converted copy numbers for the ISG‐PE were 4.2 × 10^4^, 4.4 × 10^4^, 2.8 × 10^2^, 3.2 × 10^2^ and 9.0 × 10^2^ (copies μL‐DNA^−1^) for sequencing primer sets 341F/805R, 515F/805R, *pmoA* 189f/682r, *pmoA* 189f/650r and *amoA* 1F/2R, respectively. Quantities for the qPCR were 4.1 × 10^4^, 3.4 × 10^4^, 4.0 × 10^3^, 7.4 × 10^3^ and 3.7 × 10^3^ (copies μL‐DNA^−1^) for primer sets 341F/805R, 515F/805R, *pmoA* 189f/682r, *pmoA* 189f/650r and *amoA* 1F/2R, respectively. In the case of 16S rRNA gene, quantities were 104% (341F/805R) and 136% (515F/805R) for the ISG‐PE and qPCR, respectively. In the case of functional genes, the values were 7.1, 4.4 and 24.3% for qPCR (*pmoA* 189f/682r, *pmoA* 189f/650r and *amoA* 1F/2R, respectively). The quantitation of 16S rRNA genes was comparable to that of qPCR, but the number of functional genes was underestimated.

Then, when quantified using the SE‐R1 data set, the converted copy numbers for the ISG‐SE‐R1 were 4.0 × 10^4^, 4.6 × 10^4^, 3.0 × 10^3^, 1.2 × 10^4^ and 7.8 × 10^2^ (copies μL‐DNA^−1^) for sequencing primer sets 341F/805R, 515F/805R, *pmoA* 189f/682r, *pmoA* 189f/650r and *amoA* 1F/2R, respectively. Compared to qPCR, the ISG‐SE‐R1 showed values of 97.5%, 135%, 73.9%, 164% and 21.0% for primer sets 341F/805R, 515F/805R, *pmoA* 189f/682r, *pmoA* 189f/650r and *amoA* 1F/2R, respectively. When absolute quantification was performed with SE‐R1, the quantification of the 16S rRNA gene was comparable to that of qPCR, with a minor difference as compared to PE. In addition, quantitative results for the *pmoA* gene improved in the case of SE‐R1, with quantitative results comparable to those of qPCR. However, there was a minor change in the quantity of *amoA* gene. We speculate the reason for the variability in the quantities of the *pmoA* and *amoA* genes in ISG‐PE and ISG‐SE‐R1 to be the variability in the recovery of the mock community in PE and SE‐R1, as predicted earlier (Figure S2).

### Absolute quantification of functional and 16S rRNA gene in environmental samples

**FIGURE 4:**
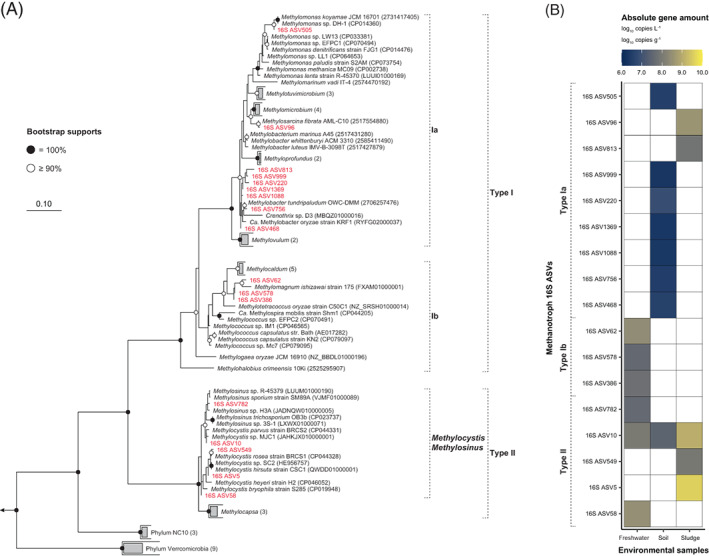
Phylogenetic analysis and absolute gene copy numbers of methanotrophs at three different environments based on 251 bp of read 1 of 16S rRNA gene amplicon sequencing. (A) Maximum likelihood tree showing the phylogenetic affiliation of methanotrophs. *Nitrosopumilus maritimus* SCM1 (GenBank accession number CP000866), *Nitrososphaera viennensis* EN76 (CP007536) and *Nitrosarchaeum* sp. AC2 (CP030847) were used as an outgroup. Circles on tree nodes indicate the confidence of branching topology with 1000 replicates, and bootstrap support values are indicated in grayscale. Sequences obtained in this study are highlighted in red. (B) Heat map representing the absolute gene copy numbers of each 16S ASV in three different environments.

**FIGURE 5:**
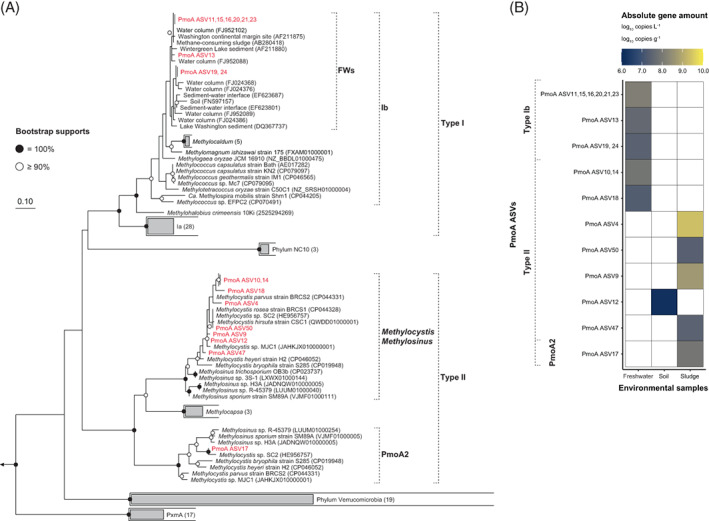
Diversity and absolute gene copy numbers of methanotrophs at three different environments based on 251 bp of read 1 of *pmoA* gene amplicon sequencing. (A) Maximum likelihood tree indicating the phylogenetic affiliation of the detected PmoA amplicon sequence variants (ASVs). The outgroup consisted of three *Nitrosomonadaceae amoA* genes (*Nitrosomonas europaea* ATCC 19718 [GenBank accession number AL954747], *Nitrosomonas ureae* strain Nm10 [CP013341] and *Nitrosospira multiformis* ATCC 25196 [CP000103]). Circles on tree nodes indicate the confidence of branching topology with 1000 replicates, and bootstrap support values are indicated in grayscale. Sequences obtained in this study are highlighted in red. (B) Heat map representing the absolute gene copy numbers of each PmoA ASV in three different environments.

To expand the applicability of the quantitative approach using synthetic ISGs, we performed quantification and phylogenetic and diversity analyses of individual ASVs in environmental samples. Amplicon sequencing was performed after PCR using five different primer sets, and the DADA2 process treated only SE‐R1 (251 bp) to obtain each dose–response curve and quantify individual ASVs (see Figure S3 for dose–response curves and linear predictors). Methanotrophs were detected in all environmental samples by 16S rRNA gene amplicon sequencing, and the diversity of methanotrophs was confirmed by *pmoA* gene amplicon sequencing. On the contrary, in the *amoA* gene amplicon sequencing, ASVs from all environmental samples were below the quantitation limit, even though the genus *Nitrosomonas*, an AOB, was among the top 10 reads in 16S rRNA gene amplicon sequencing of sludge samples (Figure S4). Herein, we performed gene quantification and phylogenetic and diversity analyses of individual methanotroph ASVs using amplicon sequencing results of the 341F/805R and *pmoA* 189f/682r primer sets whose values were close to that of the qPCR quantitative results in the mock community (Figures 4 and 5).

The 16S rRNA gene quantification and phylogenetic analysis revealed six, eight and five methanotrophic ASVs with read counts above the lower limit of the synthetic ISG in the freshwater, soil and sludge samples, respectively. The number of genes in each sample was expressed per litre of collected water, per gram of soil and per gram of sludge assuming 100% DNA extraction efficiency. The most abundant methanotrophs in these environments were 16S ASV58 (3.1 × 10^8^ copies L^−1^), 16S ASV10 (2.9 × 10^7^ copies g^−1^) and 16S ASV5 (3.8 × 10^9^ copies g^−1^) (Figure 4B), which are all closely related to the genus *Methylocystis*, a type II methanotroph (Figure 4A). Type II methanotrophs were detected and abundant in all environmental samples, whereas type Ia was detected in soil and sludge samples and type Ib in freshwater only. Particularly, most type Ia methanotrophs in the soil samples belonged to the closely related genus *Methylobacter* (Figure 4A, B).

*pmoA* gene quantification and diversity analysis revealed 12, 1 and 5 methanotroph ASVs with read counts above the lower limit of synthetic ISG in freshwater, soil and sludge, respectively. As several *pmoA* ASVs detected in freshwater had 100% sequence identity after amino acid translation, the quantitative PmoA amino acid sequence values were expressed as the sum of all *pmoA* ASV quantities and grouped together in the PmoA phylogenetic tree (Figure 4A). The freshwater sample included Type Ib and II methanotrophs, the soil sample included type II methanotrophs, and the sludge sample included type II and PmoA2 methanotrophs; the most abundant methanotrophs were the PmoA ASV11, 15, 16, 20, 21 and 23 groups (1.4 × 10^8^ copies L^−1^), PmoA ASV12 (1.6 × 10^6^ copies g^−1^) and PmoA ASV4 (2.3 × 10^9^ copies g^−1^), in these environments, respectively (Figure 4B). Type Ia methanotrophs were not detected, including ASVs below the lower limit of synthetic ISG.

## DISCUSSION

### Differences in quantitative values in the mock community among different quantification methods

Differences in the quantification method caused differences in the quantification values in the mock community. We attribute the observed differences in quantification values to the following possible reasons. We first compared qPCR to ISG‐PE and considered two reasons for the underestimation of the ISG method. First, we considered the effect of primer mismatch on the PCR amplification efficiency. The *pmoA* gene from *M. ishizawai* strain RS11D‐Pr did not have any mismatches with the *pmoA* 189f and *pmoA* 650r primers, and one mismatch with *pmoA* 682r primer. On the contrary, the *amoA* gene from *Nitrosomonas europaea* ATCC 19718 had one mismatch with the *amoA* 1F primer and none with *amoA* 2R primer. Thus, the lower amplification efficiency of these genes as compared to that of ISGs with no primer mismatch suggests that quantitative results based on amplicon sequencing, which is quantitative competitive PCR, are underestimated (Bru et al., 2008). For qPCR, the *pmoA* gene standard was obtained from the *M. ishizawai* strain RS11D‐Pr and the *amoA* gene standard from *N. europaea* ATCC 19718, which are the same as the species of bacteria used for the mock community. Therefore, the PCR amplification efficiency of the genes was the same and the number of genes was not underestimated. Bias due to primer mismatch could be improved by using primers specific to the target microorganism or group.

Second, we compared the GC content of PCR products from ISGs and microbial genes. The GC contents of all PCR products from ISGs were 50.5%–53.5%, whereas those from microbial genes were 51.5%–56.8% (341F/805R) or 54.3%–56.8% (515F/805R) for 16S rRNA gene amplicons, 63.8% (*pmoA* 189f/682r) or 63.4% (*pmoA* 189f/650r) for *pmoA* gene amplicons and 48.5% (*amoA* 1F/2R) for *amoA* gene amplicons (Table S1). In addition, it is well known that GC‐rich DNA is inefficiently amplified by PCR (Hubé et al., 2005); therefore, we examined the amplification efficiency (Figure S5). For the *pmoA* gene, the dissociation temperature was higher for the *M. ishizawai* gene than for ISG01, but there was little difference in amplification efficiency (Figure S5C and S5D). Thus, it was assumed that GC content is not a cause for underestimating quantitative sequencing because there was no noticeable difference in the amplification efficiency between the GC‐rich *M. ishizawai pmoA* gene and ISG.

Furthermore, we speculated that the reason for the large difference in quantities between ISG‐PE and ISG‐SE‐R1 in the *pmoA* gene could be attributed to a sequence‐specific error inherent to the Illumina sequencer, which is suggested to be related to the guanine‐guanine‐cytosine (GGC) sequence in the DNA template (Nakamura et al., 2011). The sequence is speculated to be a preferential inhibition factor for DNA polymerase, and incomplete extension of the template ensemble results in lagging‐strand dephasing due to preferential inhibition of the enzyme by a specific sequence of nucleic acids (Metzker, 2009; Nakamura et al., 2011). We examined the GGC sequences in the mock and ISG01‐04 microbial genes to determine whether miscalling can occur by this mechanism (Table S2). We found that the *pmoA* gene had more GGC sequences than the other genes, with more than half of them located 251 bp behind the amplicon (*pmoA* 189f/682r, 10 of 16 locations behind; *pmoA* 189f/650r, 9 of 15 locations behind). Thus, we speculated that miscalling increased as sequencing progressed, and merging behind R1 and R2, which had the most miscalls, reduced read recovery. Therefore, ISG and mock community gene reads were counted separately, and we verified non‐uniform read removal on the DADA2 processing by calculating the increment factor of SE‐R1 (251 bp) reads with respect to the PE reads (Figure S6). The analysis showed that the increment factor was significantly higher in the case of *pmoA* genes in the mock community with a concentration of miscall‐inducing sequences behind 251 bp, which confirmed our hypothesis. Thus, spiking the ISGs can be also applied to the evaluation of the read process.

In summary, when comparing the quantitative values of ISG with qPCR, we found that there are biases such as primer mismatch, sequencing quality and GGC sequence. In particular, sequencing quality and GGC sequence have a large influence when analysed by PE. However, when analysed by SE‐R1, it was found that these effects can be reduced. Therefore, if the emphasis is on quantification, analysis and quantification using the SE‐R1 data set are effective. Additionally, when using the SE‐R1 data set, our results indicate that synthetic ISGs are useful not only for quantifying total microbial abundance and 16S rRNA gene copy number for individual taxa (Figure S7) but also for quantifying the *pmoA* gene as a functional marker (Figure 3C).

### Comparison of microbial phylogeny, functional diversity and quantities

Quantitative sequencing in the *amoA* gene showed that ASVs from all environmental samples were below the lower limit of quantification. The *amoA* gene was not detected in the sludge samples, even though 16S ASV6 (3.0 × 10^9^ copies g^−1^), classified as the genus *Nitrosomonas* in the sludge samples, was the third most abundant microorganism overall in the sludge sample (Figure S4). It has been reported that the *amoA* 1F/2R primer set used in this study has a low coverage for the specific AOB cluster *amoA* gene, while a primer set targeting the 16S rRNA gene has a wide coverage (Dechesne et al., 2016). It was suggested that the AOB lineage quantified based on the 16S rRNA gene in this study is rarely detected in *amoA* 1F/2R or has lower coverage than the 16S rRNA gene (Figure S8). Thus, differences in primer coverage are a serious problem in quantitative sequencing with spiking ISGs, which is part of the quantitative competitive PCR assay.

Type Ia methanotrophs, which are relatively rare in the 16S rRNA gene base, were difficult to detect in the *pmoA* gene base quantitative sequencing. This may be an underestimation because the *pmoA* 189f/682r primer set is not compatible with every type I methanotrophs (Holmes et al., 1995; Reay et al., 2001). Thus, considering that absolute quantities based on the *pmoA* gene may be underestimated, a comparison of ASV positions and quantities in the two phylogenetic trees may predict ASVs attributable to the same species. As an example, type II methanotroph 16S ASV5 (3.8 × 10^9^ copies g^−1^) and PmoA ASV4 (2.3 × 10^9^ copies g^−1^) in the sludge sample are linked based on their tree position and quantities (Figures 4 and 5). These ASVs (16S ASV5 and PmoA ASV4) showed 6.1 × 10^9^ copies g^−1^ and 3.5 × 10^9^ copies g^−1^ by qPCR, respectively, and the quantitative sequencing values were 61.9 and 65.5% of the qPCR values (16S rRNA and *pmoA* gene, respectively) (Figure S9) (see ‘Methods and Results’ in Data S1 for details). Based on rrnDB database, type II methanotrophs *Methylocystis* and *Methylosinus* contain 1~3 copies of the 16S rRNA gene per genome (using rrnDB v5.8 [web‐search performed on 29 September 2022]) (Stoddard et al., 2015). In addition, type II methanotrophs may possess 1~3 copies of the *pmoA* gene per cell (Auman et al., 2000); therefore, if we allow for a maximum error of three times, we emphasize that one species possesses 16S ASV5 and PmoA ASV4 in the genome. 16S ASV10 (1.2 × 10^9^ copies g^−1^) and PmoA ASV9 (4.4 × 10^8^ copies g^−1^) are also inferred to be located on the same genome based on tree position and quantitative values (Figures 4 and 5). In case of the freshwater sample, PmoA ASV quantification values for type Ib methanotroph were 2.6 × 10^7^–1.4 × 10^8^ copies L^−1^ and 16S ASV quantification values were 4.0 × 10^7^–2.7 × 10^8^ copies L^−1^. Based on these results, it seems possible to estimate the approximate phylogenetic position of the 16S rRNA gene in uncultured freshwater clusters of PmoA type Ib methanotrophs (Figures 4 and 5). 16S ASV10 (2.9 × 10^7^ copies g^−1^) and PmoA ASV12 (1.6 × 10^6^ copies g^−1^) in the soil sample were the only type II methanotrophs, but their quantitative values had more than a 10‐fold difference (Figures 4 and 5). In addition, 16S ASV10 was inferred to be linked to PmoA ASV9, but it was missed in the soil sample. The possible reasons for this are that there was a slight difference in PCR amplification efficiency between PmoA ASV9 and PmoA ASV12, which resulted in a detection limit below the limit of detection, and furthermore, PmoA ASV9 and PmoA ASV12 are closely related and cannot be distinguished by the 16S rRNA gene length of 251 bp and may be encapsulated in a single 16S ASV10. Overall, quantitative sequencing of phylogenetic and functional marker genes presents the possibility of a more robust inference of phylogenetic and functional relationships in major microbial species with no primer mismatches, although the quantitative values are biased by the mismatches used in the design step of synthetic ISGs.

## CONCLUSIONS

Quantities of 16S rRNA and *pmoA* genes using the quantitative sequencing method with synthetic ISGs are equivalent to those obtained by real‐time qPCR in the mock community. In environmental samples, we successfully quantified individual ASV of both 16S rRNA and *pmoA* genes and inferenced phylogenetic and functional relationships. This is a unique and highly scalable method that can change the target genes by simply changing the primers used in the design stage of the synthetic ISGs. This method will promote the study of microbial dynamics in natural and engineered systems by simplifying the design of research methods that combine phylogenetic, functional diversity and quantitative analysis of microorganisms.

## AUTHOR CONTRIBUTIONS

**Kazuyoshi Koike:** Data curation (equal); formal analysis (lead); investigation (lead); methodology (supporting); visualization (lead); writing – original draft (lead). **Ryo Honda:** Resources (equal); supervision (supporting); writing – review and editing (equal). **Masataka Aoki:** Resources (equal); writing – review and editing (equal). **Ryoko Yamamoto‐Ikemoto:** Resources (equal); supervision (supporting). **Kazuaki Syutsubo:** Project administration (supporting); resources (equal); writing – review and editing (supporting). **Norihisa Matsuura:** Conceptualization (lead); data curation (lead); funding acquisition (lead); methodology (lead); project administration (equal); resources (lead); software (lead); supervision (lead); validation (equal); writing – review and editing (lead).

## FUNDING INFORMATION

Leading Initiative for Excellent Young Researchers from the MEXT; JSPS KAKENHI, Grant Number: 22K19866; JST SPRING, Grant Number: JPMJSP2135.

## CONFLICT OF INTEREST STATEMENT

The authors declare that there is no conflict of interest.

## Supporting information


**Data S1.** Supporting Information.

## Data Availability

Illumina MiSeq raw data of the mock community and environmental samples to which ISGs were added are deposited in the DDBJ Sequence Read Archive (DRA) under the accession codes DRA014916 and DRA014915, respectively.
