# Supplementary material for: A quantitative sequencing method using synthetic internal standards including functional and phylogenetic marker genes
Source: Environ Microbiol Rep. 2023 Jul 18;15(6):497–511. doi: 10.1111/1758-2229.13189 (PMC10667660; doi:10.1111/1758-2229.13189)
Supplement: Supplementary file 1 — Data S1. Supporting Information. [file EMI4-15-497-s001.docx]

**Supplemental** **Material**

**A quantitative sequencing method using synthetic internal standards including functional and phylogenetic marker genes**

Kazuyoshi Koike^1^, Ryo Honda^2^, Masataka Aoki^3^, Ryoko Yamamoto-Ikemoto^2^, Kazuaki Syutsubo^3,4^, Norihisa Matsuura^2^

^1^ Graduate School of Natural Science and Technology, Kanazawa University, Kakuma, Kanazawa, 920-1192, Japan

^2^ Faculty of Geosciences and Civil Engineering, Kanazawa University, Kakuma, Kanazawa, 920-1192, Japan

^3^ Regional Environment Conservation Division, National Institute for Environmental Studies (NIES), Onogawa, Tsukuba, Ibaraki 305-8506, Japan

^4^ Research Center for Water Environment Technology, School of Engineering, the University of Tokyo, Bunkyo, Tokyo 113-8656, Japan

**Correspondence**

Norihisa Matsuura, Faculty of Geosciences and Civil Engineering, Kanazawa University, Kakuma, Kanazawa, 920-1192, Japan

Email: matsuura@se.kanazawa-u.ac.jp

This supporting information contains 30 pages, 2 methods, 1 result, 9 figures and 5 tables.

**Supplementary Methods**

*Genomic DNA extraction and purification*

Genomic DNA extraction was performed according to the following protocol: each fully grown culture (*Methylomagnum ishizawai*; 20 mL, *Nitrosomonas europaea*; 50 mL, and *Escherichia coli*; 10 mL) was separately centrifuged (12,000 *g*, 10 min, 4 ºC) and the pellets were washed once with 1× phosphate-buffered saline (PBS) (12,000 *g*, 10 min, 4 ºC). The washed pellets were resuspended in 800 µL pH 8.0 Tris-EDTA (TE) buffer (NIPPON GENE, Japan), and 800 µL phenol (pH 8.0) (NIPPON GENE, Japan) was added. To avoid shearing of the genomic DNA, the tubes were gradually inverted many times during the extraction process. After inverting, samples were centrifuged (15,000 *g*, 5 min, 21 ºC), and 750 µL of the supernatant was extracted with an equal volume of phenol/chloroform/isoamyl alcohol (PCI; 25:24:1 v/v; NIPPON GENE, Japan). Subsequently, 700 µL of the supernatant (15,000 *g*, 5 min, 21 ºC) was extracted using an equal volume of PCI. After complete protein removal, 650 µL of the supernatant (15,000 g, 5 min, 21 ºC) was extracted using an equal volume of chloroform/isoamyl alcohol (CIA; 24:1, v/v; NIPPON GENE, Japan). Then, 600 µL of the supernatant (15,000 *g*, 5 min, 21 ºC) was extracted with an equal volume of CIA. The aqueous phase (15,000 *g*, 5 min, 21 ºC) was transferred to a fresh tube and the nucleic acid was precipitated for at least 30 min at −20 ºC with 0.1 volumes of 3 M sodium acetate (pH 5.2) (FUJIFILM Wako Pure Chemical, Japan) and 2.5 volumes of ethanol (FUJIFILM Wako Pure Chemical, Japan). After centrifugation (20,000 g, 30 min, 4 ºC), the nucleic acid pellets were washed once with 1 mL of ice-cold 70% ethanol, further centrifuged (20,000 g, 30 min, 4 ºC), dried at room temperature for 2-5 min, and then dissolved in 50 µL of UltraPure™ DNase/RNase-Free Distilled Water (Invitrogen, USA).

Genomic DNA extracts were treated with deoxyribonuclease-free ribonuclease (RNase) in glycerol solution (NIPPON GENE, Japan) according to the following protocol to remove any RNA contamination: reaction mixture (100 µL) containing 0.2 µL (20 ng µL-1) of RNase A glycerol solution, 30µL of DNA extracts, and 69.8 µL of TE buffer (pH 8.0), and incubated at 37°C for 20 min. RNase A inactivation and pure DNA recovery were performed using PCI treatment and ethanol precipitation according to the following protocol: 300 µL of UltraPure™ DNase/RNase-Free Distilled Water was added to obtain a total volume of 400 µL. The subsequent purification procedure followed the initial DNA purification protocol described above, including PCI and CIA treatment, and ethanol precipitation, with starting volumes of 400, 380, and 350 µL, respectively. The resulting DNA precipitates were dissolved in 50 µL of UltraPure™ DNase/RNase-Free distilled water, stored at 4 ºC, and immediately used in the next step.

*Quantitative real-time PCR of the sludge sample*

The numbers of *pmoA*, *amoA*, and 16S rRNA genes in the sludge sample were measured by quantitative real-time PCR (qPCR) assay, using primers without Illumina adapter sequences (primer sequences and thermocycling protocols in Table 1). All DNA extracts were handled using Axygen Maxymum Recovery products (CORNING, USA) to minimize DNA loss in microtubes and pipette tips. For the functional gene standard preparation, the genomic DNA of *M. ishizawai* and *N. europaea* were used to amplify *pmoA* and *amoA* genes, respectively, using primers with Illumina adapter sequences attached to the 5' end of the forward and reverse primers (Table S6). Approximately full-length 16S rRNA gene of *E. coli* was amplified using primers without Illumina adapter sequences (Table S6) for its gene standard preparation. Each standard curve was prepared from a 10-fold serial dilution of the amplified gene (4.0×10^6^-10^2^ copies µL^-1^). All qPCR assays were carried out on an Agilent Technologies Stratagene Mx3000P Real-Time qPCR system (Agilent Technologies, USA) in a total volume of 20 µL containing 1× QuantiTect SYBR Green PCR Master Mix (QIAGEN, Germany), 200 nM of forward and reverse primers each (synthesized by Eurofins Genomics, Japan) and 2.0 µL of DNA template. All reactions were performed in biological triplicate (n=3). Ct values were obtained using the MxPro qPCR Software (Agilent Technologies, USA), and gene copy numbers were calculated using calibration curves.

The calculated number of gene copies per unit volume of each reaction was converted to that of DNA used for qPCR using equation (i):

$$Z_{gene conc.}\text{ = }\frac{\frac{Y_{gene conc.} \times D_{sample dilution vol.}}{E_{diluted vol.}} \times H_{qPCR use vol.}}{I_{qPCR reaction vol.}}$$

where *Z_gene conc._* (copies µL-reaction^-1^) is the qPCR gene concentration using the linear predictor, *Y_gene conc._* (copies µL-DNA^-1^) is the gene concentration in the sample after DNA extraction, *D_sample dilution vol._* (µL) is the volume of DNA used for dilution, *E_diluted vol._* (µL) is the volume of DNA after dilution, *H_qPCR use_ _vol._* (µL) is the volume of the sample used for qPCR, and *I_qPCR reaction vol._* (µL) is the qPCR reaction volume. Additionally, the number of genes per DNA volume of sludge sample was converted to the number of genes per sample weight assuming 100% DNA extraction efficiency using the following equation (ii):

$$X_{gene conc.}\text{ = }\frac{Y_{gene conc.} \times C_{DNA extraction vol.}}{A_{sample wt. or vol.}}$$

where *X_gene conc._* (copies g^-1^) is the assumed gene concentration in the soil sample, *Y_gene conc._* (copies µL-DNA^-1^) is the gene concentration in the sample after DNA extraction, *C_DNA extraction vol._* (µL) is the DNA concentration of the sample, and *A_sample wt. or vol._* (g or L) is the sample weight or volume.

In addition, to determine the gene abundance of individual ASVs, we took the fractional abundance of the ASVs (percent reads per sample calculated by excluding the number of ISG reads from the ISG-spiked sequencing data) and multiply this by the total concentration of those genes determined via qPCR.

**Supplementary Results**

*Pearson's correlation between PE read recovery ratio and amplicon length*

Illumina sequencing quality regresses toward the end of the reads, and low-quality reverse reads reduce the read recovery ratio after merging (Ramakodi, 2021). In this study, we examined Pearson's correlation between amplicon length and PE read recovery ratio. As expected, a strong negative Pearson's correlation (*r* = −0.976) between the PE read-recovery ratio and amplicon length was found (Figure S1).

*Quantitative values of the sludge sample*

To assess the quantitative value of quantitative sequencing using synthetic ISGs in environmental samples, qPCR of 16S rRNA, *pmoA*, *amoA* gene was performed. After that, each ASV copies were calculated using relative percentage based on the amplicon sequencing. First, the *amoA* gene was below the quantification limit by qPCR as well as by quantitative sequencing. The gene amounts of 16S ASVs classified as methanotrophs was 6.1×10^9^, 1.9×10^9^, 6.2×10^8^, 1.5×10^8^, and 1.2×10^8^ copies g^-1^, 16S ASV5, 10, 96, 549, and 813, respectively (Figure 6). The quantitative sequencing values were 61.9% of qPCR, which tended to underestimate the results tested in the mock community. The qPCR quantification value for PmoA ASVs were 3.5×10^9^, 6.7×10^8^, 1.3×10^8^, 4.1×10^7^, 3.6×10^7^ copies g^-1^, PmoA ASV4, 9, 17, 47, 50, respectively (Figure 6). The quantitative sequencing values were 65.5% of qPCR, which was almost equivalent to the results tested in the mock community. These results show that quantitative sequencing provides results comparable to qPCR even in environmental samples.

**Figure S1:** Pearson's correlation (*r*) between PE read recovery ratio and amplicon length.

**Figure S2:** Comparison of read recovery ratio before and after DADA2 processing of mock community genes with paired-end (PE) datasets and single-end (SE) 251 bp forward (R1) datasets.

**Figure S3:** Internal standard curve for absolute quantification of environmental samples drawn by negative binomial generalized linear model regression analysis of the dose-response curve with the slope of the model fixed at 1.

**Figure S4:** Ranking of the top 10 abundant amplicon sequence variants (ASVs) in absolute quantified gene copy number based on 16S rRNA gene amplicon sequencing with primer set 341F/805R. Taxonomy were indicated by p, phylum; c, class; o, order; f, family; g, genus.

**Figure S5:** Comparison of one of the mock community species and ISG01 at the same DNA template concentration. Graphs showing amplification plots and dissociation curves of *E. coli*, *M. ishizawai*, *N. europaea*, and ISG01 amplified with each primer sets. (A) *E. coli* 16S rRNA gene amplification with 341F/805R primers. (B) *E. coli* 16S rRNA gene amplification with 515F/805R primers. (C) *M. ishizawai* *pmoA* gene amplification with *pmoA* 189f/682r primers. (D) *M. ishizawai* *pmoA* gene amplification with *pmoA* 650r/682r primers. (E) *N. europaea amoA* gene amplification with *amoA* 1F/2R primers.

**Figure S6:** Graph showing the significant increase in the read increment factor of SE-R1 with respect to the PE of the DADA2 process in *pmoA* genes. The increment factor was calculated by dividing the number of SE-R1 reads by the number of PE reads.

**Figure S7:** Bar graph of 16S rRNA gene copy number for individual taxa using synthetic internal standard genes. The qPCR is shown as the mean value of three replicated experiments.

**Figure S8:** Phylogenetic analysis and absolute gene copy numbers of AOBs at three different environments based on 251 bp of read 1 of 16S rRNA gene amplicon sequencing. (A) Maximum likelihood tree showing the phylogenetic affiliation of AOBs. *Denitromonas halophilus* SFB-3 k141_27 (GenBank accession number VMNK01000025) and *Denitromonas indolicum* MPKc (AY972852) were used as an outgroup. Circles on tree nodes indicate the confidence of branching topology with 1000 replicates, and bootstrap support values are indicated in grayscale. Sequences obtained in this study are highlighted in red. (B) Heat map representing the absolute gene copy numbers of each 16S ASV in three different environments.

**Figure S9:** Quantitative value comparison of methanotrophs in the sludge between the quantitative sequencing using synthetic ISGs and qPCR. SE-R1 was used for quantitative sequencing. (A) Comparison of quantitative values of 16S rRNA gene amplified by primer set 341F/805R. (B) Comparison of quantitative values of *pmoA* gene amplified by primer set *pmoA* 189f/682r.

**Table S1:** GC contents of the mock community.

| Target | Primer set | GC content (%) | Product length (bp) |
| --- | --- | --- | --- |
| ISG01 | 341F/805R | 52.3 | 465 |
| ISG01 | 515F/805R | 50.5 | 291 |
| ISG01 | *pmoA* 189f/682r | 53.1 | 531 |
| ISG01 | *pmoA* 189f/650r | 53.2 | 500 |
| ISG01 | *amoA* 1F/2R | 52.1 | 491 |
| ISG02 | 341F/805R | 51.4 | 465 |
| ISG02 | 515F/805R | 50.9 | 291 |
| ISG02 | *pmoA* 189f/682r | 51.8 | 531 |
| ISG02 | *pmoA* 189f/650r | 52.0 | 500 |
| ISG02 | *amoA* 1F/2R | 51.3 | 491 |
| ISG03 | 341F/805R | 51.2 | 465 |
| ISG03 | 515F/805R | 51.9 | 291 |
| ISG03 | *pmoA* 189f/682r | 51.8 | 531 |
| ISG03 | *pmoA* 189f/650r | 51.8 | 500 |
| ISG03 | *amoA* 1F/2R | 50.7 | 491 |
| ISG04 | 341F/805R | 52.9 | 465 |
| ISG04 | 515F/805R | 51.2 | 291 |
| ISG04 | *pmoA* 189f/682r | 53.5 | 531 |
| ISG04 | *pmoA* 189f/650r | 53.4 | 500 |
| ISG04 | *amoA* 1F/2R | 52.3 | 491 |
| *Escherichia coli* NBRC 3301 | 341F/805R | 55.1 | 465 |
| *Methylomagnum ishizawai* strain RS11D-Pr | 341F/805R | 56.8 | 465 |
| *Nitrosomonas europaea* ATCC 19718 | 341F/805R | 56.8 | 465 |
| *Escherichia coli* NBRC 3301 | 515F/805R | 56.0 | 291 |
| *Methylomagnum ishizawai* strain RS11D-Pr | 515F/805R | 56.8 | 291 |
| *Nitrosomonas europaea* ATCC 19718 | 515F/805R | 54.3 | 291 |
| *Methylomagnum ishizawai* strain RS11D-Pr | *pmoA* 189f/682r | 63.8 | 531 |
| *Methylomagnum ishizawai* strain RS11D-Pr | *pmoA* 189f/650r | 63.4 | 500 |
| *Nitrosomonas europaea* ATCC 19718 | *amoA* 1F/2R | 48.5 | 491 |

**Table S2:** Location of GGC sequences in the amplicon sequence, excluding primer sequences. The 251 bp region of the forward read (R1) is marked in red and the GGC sequence in yellow.

16S rRNA gene amplicon sequence (341F/805R) (5**'**-3**'**)

>ISG01

CGAAGTCTTGGGGGTTTCTACTGGTGGTACGTGATCAGCCGTGCCTACGTCGAGCTACTACCACACTCTCGGCTCAATTACCGTGTGACATCGGATACTCCAACATGGCACGGCGACTGCTAGATGCGAGAAGCCTTACGTCTGCATAGACAGGTTAGTGCCAGCAGCCGCGGTAATTCCGATGACCTGCTTACCATGTTATAGCACCTCGTTTAGCAGCATGACGCAACTCGAGAGTCGCATTTGCGTAAACGGATACCATAGTGAAGTGCACATTCACGAAGTCCCTACCATAGAGCGGAATGTTCTAGAAGCCCGTTCGCAACACCTTAGGCCTATGGTTGAATTCACCGGTGTGGTCTCTGCACAACCTTCGGTAAGGATGTAATCCATGGCTAGGCGTTCTTCTCAGCGTTCTGTTCCATGT

>ISG02

CGCCTTATACGGGGTTTCTACTGGTGGTTTCTCGGTTCATGGATCCACCTGTTACTTGCGGCTTGCCCACAAACGGTCCCTAGCGTTAGCCTTTGAAGAGAATAGATCACCTCCTATTATGGAATGAGCCGATCCTATGCTCATAGCCTGCACTCTGGTGCCAGCAGCCGCGGTAATCATGAAGGGTAATACGGGAAGACTAGTGACGGGTAAACTCCTTAGGACACAACTCACGTCAACCGCCGTTCAGATAGATAGGCCCTGTCGTCATACGTTCGCAAATGTTCCTTGGTACCCGGGTCATCATAGTCTGTTTGGGTAAACTGAACCGGCCAATCTCCTGAACAGACAAATCCGCCTCGCATATCTACCTTCGGTAAGGATGTTCGAATACCTCTAGCGTTCTTCTCAGCGTTCGTCCGCTTCA

>ISG03

TTGTGCTGCAGGGGTTTCTACTGGTGGTGTATAGCTACTATAGCGTAGGGATCGATATCAGCTATACCTAGATGAGAGCCCATTTCCGCTCGATATACCTAGGGACACGTAGATGTACCCTCTAGCAAGAGTTTGCCACAGATACGTACCGCTCATAGTGCCAGCAGCCGCGGTAAACGCGAACCGAAGCGCAGTAGAAGTACTCCGTATCCTACCTCGGTCGTGGTTTAGGCTATCGACATCTTGCATGGGCTTCCCTAGTGAACTCTACCCTTGTAACCTACACTGGTGTAAGACATCAGACAACGAACGCTAACTATCCTGCAACACTCCGGAAGAGATGAAGTCGCCGACGCGATACGAAACTTCACCTTCGGTAAGGATGTTTACGCTAGGACTGCGTTCTTCTCAGCGTTCGAGCATCTAC

>ISG04

GGAAGTAGAGGGGGTTTCTACTGGTGGTTTACCGGACGACCGATGCATAGAACCGCTTATTCGCGGTACAAGTTGTGATGACGGAAACCTACGTCGAACGAAAGCGGCTAGCCCTTGGAACGCACTCTCGGATGTACCACACCAGACATGCGCCGTTGTGCCAGCAGCCGCGGTAAATGGATTATCAGGTTAGCCCAACAGGTTCGGTACATGTCGCTAGCTTGACTGGCCTAGGATCTCGAGATATCGGTGCACATCGTACCGAGATCACGCGAAATACTGCACTTACAGTAGCCATAGTCAGCATTAACGGGCGCATAAACTTACCGAGGAACCTTAAACGACACTGCGCTAAACGCTAGTGCGTCAACCTTCGGTAAGGATGTTCCGGCTTCATTCGCGTTCTTCTCAGCGTTCGTATGTCCGA

>*Escherichia coli* NBRC 3301

TGGGGAATATTGCACAATGGGCGCAAGCCTGATGCAGCCATGCCGCGTGTATGAAGAAGGCCTTCGGGTTGTAAAGTACTTTCAGCGGGGAGGAAGGGAGTAAAGTTAATACCTTTGCTCATTGACGTTACCCGCAGAAGAAGCACCGGCTAACTCCGTGCCAGCAGCCGCGGTAATACGGAGGGTGCAAGCGTTAATCGGAATTACTGGGCGTAAAGCGCACGCAGGCGGTTTGTTAAGTCAGATGTGAAATCCCCGGGCTCAACCTGGGAACTGCATCTGATACTGGCAAGCTTGAGTCTCGTAGAGGGGGGTAGAATTCCAGGTGTAGCGGTGAAATGCGTAGAGATCTGGAGGAATACCGGTGGCGAAGGCGGCCCCCTGGACGAAGACTGACGCTCAGGTGCGAAAGCGTGGGGAGCAAACA

>*Methylomagnum ishizawai* strain RS11D-Pr

TGGGGAATATTGGACAATGGGCGCAAGCCTGATCCAGCAATGCCGCGTGTGTGAAGAAGGCCTGCGGGTTGTAAAGCACTTTAAGCAGGAAAGAAGGCTCCAAGGCCAATACCCTTGGAGATTGACGTTACCTGCAGAATAAGCACCGGCTAACTCCGTGCCAGCAGCCGCGGTAATACGGAGGGTGCGAGCGTTAATCGGAATTACTGGGCGTAAAGCGCGCGTAGGCGGTCCGTTAAGTCAGCCGTGAAAGCCCCGGGCTTAACCTGGGAACTGCGGATGATACTGGCGGACTAGAGTGTGGCAGAGGGTGGCGGAATTTCCGGTGTAGCAGTGAAATGCGTAGAGATCGGAAGGAACACCAGTGGCGAAGGCGGCCATCTGGGCCAACACTGACGCTGAGGTGCGAAAGCGTGGGGAGCAAACA

>*Nitrosomonas europaea* ATCC 19718

TGGGGAATTTTGGACAATGGGCGAAAGCCTGATCCAGCCATGCCGCGTGAGTGAAGAAGGCCTTCGGGTTGTAAAGCTCTTTTAGTCGGAAAGAAAGAGTTGCAATGAATAATTGTGATTTATGACGGTACCGACAGAAAAAGCACCGGCTAACTACGTGCCAGCAGCCGCGGTAATACGTAGGGTGCGAGCGTTAATCGGAATTACTGGGCGTAAAGGGTGCGCAGGCGGTCTTGCAAGTCAGATGTGAAAGCCCCGGGCTTAACCTGGGAATTGCGTTTGAAACTACAAGGCTAGAGTGCAGCAGAGGGGAGTGGAATTCCATGTGTAGCAGTGAAATGCGTAGAGATGTGGAAGAACACCGATGGCGAAGGCAGCTCCCTGGGTTGACACTGACGCTCATGCACGAAAGCGTGGGGAGCAAACA

16S rRNA gene amplicon sequence (515F/805R) (5**'**-3**'**)

>ISG01

TTCCGATGACCTGCTTACCATGTTATAGCACCTCGTTTAGCAGCATGACGCAACTCGAGAGTCGCATTTGCGTAAACGGATACCATAGTGAAGTGCACATTCACGAAGTCCCTACCATAGAGCGGAATGTTCTAGAAGCCCGTTCGCAACACCTTAGGCCTATGGTTGAATTCACCGGTGTGGTCTCTGCACAACCTTCGGTAAGGATGTAATCCATGGCTAGGCGTTCTTCTCAGCGTTCTGTTCCATGT

>ISG02

TCATGAAGGGTAATACGGGAAGACTAGTGACGGGTAAACTCCTTAGGACACAACTCACGTCAACCGCCGTTCAGATAGATAGGCCCTGTCGTCATACGTTCGCAAATGTTCCTTGGTACCCGGGTCATCATAGTCTGTTTGGGTAAACTGAACCGGCCAATCTCCTGAACAGACAAATCCGCCTCGCATATCTACCTTCGGTAAGGATGTTCGAATACCTCTAGCGTTCTTCTCAGCGTTCGTCCGCTTCA

>ISG03

ACGCGAACCGAAGCGCAGTAGAAGTACTCCGTATCCTACCTCGGTCGTGGTTTAGGCTATCGACATCTTGCATGGGCTTCCCTAGTGAACTCTACCCTTGTAACCTACACTGGTGTAAGACATCAGACAACGAACGCTAACTATCCTGCAACACTCCGGAAGAGATGAAGTCGCCGACGCGATACGAAACTTCACCTTCGGTAAGGATGTTTACGCTAGGACTGCGTTCTTCTCAGCGTTCGAGCATCTAC

>ISG04

ATGGATTATCAGGTTAGCCCAACAGGTTCGGTACATGTCGCTAGCTTGACTGGCCTAGGATCTCGAGATATCGGTGCACATCGTACCGAGATCACGCGAAATACTGCACTTACAGTAGCCATAGTCAGCATTAACGGGCGCATAAACTTACCGAGGAACCTTAAACGACACTGCGCTAAACGCTAGTGCGTCAACCTTCGGTAAGGATGTTCCGGCTTCATTCGCGTTCTTCTCAGCGTTCGTATGTCCGA

>*Escherichia coli* NBRC 3301

TACGGAGGGTGCAAGCGTTAATCGGAATTACTGGGCGTAAAGCGCACGCAGGCGGTTTGTTAAGTCAGATGTGAAATCCCCGGGCTCAACCTGGGAACTGCATCTGATACTGGCAAGCTTGAGTCTCGTAGAGGGGGGTAGAATTCCAGGTGTAGCGGTGAAATGCGTAGAGATCTGGAGGAATACCGGTGGCGAAGGCGGCCCCCTGGACGAAGACTGACGCTCAGGTGCGAAAGCGTGGGGAGCAAACA

>*Methylomagnum ishizawai* strain RS11D-Pr

TACGGAGGGTGCGAGCGTTAATCGGAATTACTGGGCGTAAAGCGCGCGTAGGCGGTCCGTTAAGTCAGCCGTGAAAGCCCCGGGCTTAACCTGGGAACTGCGGATGATACTGGCGGACTAGAGTGTGGCAGAGGGTGGCGGAATTTCCGGTGTAGCAGTGAAATGCGTAGAGATCGGAAGGAACACCAGTGGCGAAGGCGGCCATCTGGGCCAACACTGACGCTGAGGTGCGAAAGCGTGGGGAGCAAACA

>*Nitrosomonas europaea* ATCC 19718

TACGTAGGGTGCGAGCGTTAATCGGAATTACTGGGCGTAAAGGGTGCGCAGGCGGTCTTGCAAGTCAGATGTGAAAGCCCCGGGCTTAACCTGGGAATTGCGTTTGAAACTACAAGGCTAGAGTGCAGCAGAGGGGAGTGGAATTCCATGTGTAGCAGTGAAATGCGTAGAGATGTGGAAGAACACCGATGGCGAAGGCAGCTCCCTGGGTTGACACTGACGCTCATGCACGAAAGCGTGGGGAGCAAACA

*pmoA* gene amplicon sequence (*pmoA* 189f/682r) (5**'**-3**'**)

>ISG01

GCACGGAAATAAGACGTGCCGACAAATTCCTCTTATGACCAAAGTGGGCGTCCATGGCTTAGACTCGTGTGGCTCGAACCCTACGGGAGGCAGCAGCGAAGTCTTGGGGGTTTCTACTGGTGGTACGTGATCAGCCGTGCCTACGTCGAGCTACTACCACACTCTCGGCTCAATTACCGTGTGACATCGGATACTCCAACATGGCACGGCGACTGCTAGATGCGAGAAGCCTTACGTCTGCATAGACAGGTTAGTGCCAGCAGCCGCGGTAATTCCGATGACCTGCTTACCATGTTATAGCACCTCGTTTAGCAGCATGACGCAACTCGAGAGTCGCATTTGCGTAAACGGATACCATAGTGAAGTGCACATTCACGAAGTCCCTACCATAGAGCGGAATGTTCTAGAAGCCCGTTCGCAACACCTTAGGCCTATGGTTGAATTCACCGGTGTGGTCTCTGCACAACCTTCGGTAAGGATGTAATCCATGGCTAG

>ISG02

ATCACCTTCAAACTGGTCCAAGGTACACTAGGACGACGCGTCATTTGGGATTGGTTACGAAGGATGCCCTCACACCCATCCTACGGGAGGCAGCAGCGCCTTATACGGGGTTTCTACTGGTGGTTTCTCGGTTCATGGATCCACCTGTTACTTGCGGCTTGCCCACAAACGGTCCCTAGCGTTAGCCTTTGAAGAGAATAGATCACCTCCTATTATGGAATGAGCCGATCCTATGCTCATAGCCTGCACTCTGGTGCCAGCAGCCGCGGTAATCATGAAGGGTAATACGGGAAGACTAGTGACGGGTAAACTCCTTAGGACACAACTCACGTCAACCGCCGTTCAGATAGATAGGCCCTGTCGTCATACGTTCGCAAATGTTCCTTGGTACCCGGGTCATCATAGTCTGTTTGGGTAAACTGAACCGGCCAATCTCCTGAACAGACAAATCCGCCTCGCATATCTACCTTCGGTAAGGATGTTCGAATACCTCTA

>ISG03

CAGTGAAAGGGATGTGCTTGATACCGTGGGTATACCTCCGTGGTAATAAGCACCGTAATAAGTGAGTCCCGCGGGCTTACCTACGGGAGGCAGCAGTTGTGCTGCAGGGGTTTCTACTGGTGGTGTATAGCTACTATAGCGTAGGGATCGATATCAGCTATACCTAGATGAGAGCCCATTTCCGCTCGATATACCTAGGGACACGTAGATGTACCCTCTAGCAAGAGTTTGCCACAGATACGTACCGCTCATAGTGCCAGCAGCCGCGGTAAACGCGAACCGAAGCGCAGTAGAAGTACTCCGTATCCTACCTCGGTCGTGGTTTAGGCTATCGACATCTTGCATGGGCTTCCCTAGTGAACTCTACCCTTGTAACCTACACTGGTGTAAGACATCAGACAACGAACGCTAACTATCCTGCAACACTCCGGAAGAGATGAAGTCGCCGACGCGATACGAAACTTCACCTTCGGTAAGGATGTTTACGCTAGGACT

>ISG04

TCTCGCATGCATTCCGGCTCGGTAGTTACGGGTCGCGCATAAGAGTCGTACCGAAGCATTCCGTTATCCTAGGAACAAACCTACGGGAGGCAGCAGGGAAGTAGAGGGGGTTTCTACTGGTGGTTTACCGGACGACCGATGCATAGAACCGCTTATTCGCGGTACAAGTTGTGATGACGGAAACCTACGTCGAACGAAAGCGGCTAGCCCTTGGAACGCACTCTCGGATGTACCACACCAGACATGCGCCGTTGTGCCAGCAGCCGCGGTAAATGGATTATCAGGTTAGCCCAACAGGTTCGGTACATGTCGCTAGCTTGACTGGCCTAGGATCTCGAGATATCGGTGCACATCGTACCGAGATCACGCGAAATACTGCACTTACAGTAGCCATAGTCAGCATTAACGGGCGCATAAACTTACCGAGGAACCTTAAACGACACTGCGCTAAACGCTAGTGCGTCAACCTTCGGTAAGGATGTTCCGGCTTCATTC

>*Methylomagnum ishizawai* strain RS11D-Pr

TCCGACTGGAAGGACCGCCGCCTGTGGGTCACCGTGACCCCGATCGTGCTGGTGACCTTCCCGGCCGCCGCGCAGGTGTTCCTGTGGGAACGTTTCCGCCAGCCGTGGGCCGCGACCGTGTGCGTGTTGGCGCTGCTGTTCGGTGAATGGGTCAACCGCTACTTCAACTTCTGGGGCTGGACCTACTTCCCCGTGAACTTCGTGTTCCCGGCCATCCTGGTCCCGGGCGCGATCATCCTCGACGTCTGCCTGATGCTGTCCGGCAGCTACCTGTTCACCGCCATCATCGGTGGCCTGGCCTGGGGTTTGATCTTCTACCCCGGCAACTGGCCGATCATCGCCCCGATCCACGTGCCGGTTGAATACAACGGCATGCTGATGTCCATCGCCGACATCCAGGGCTACAACTATGTGCGTACCGGCACCCCCGAGTACATCCGCATGGTCGAGAAGGGCACCCTGCGTACCTTCGGTAAGGACGTGGCCCCGGTGTCC

*pmoA* gene amplicon sequence (*pmoA* 189f/650r) (5**'**-3**'**)

>ISG01

GCACGGAAATAAGACGTGCCGACAAATTCCTCTTATGACCAAAGTGGGCGTCCATGGCTTAGACTCGTGTGGCTCGAACCCTACGGGAGGCAGCAGCGAAGTCTTGGGGGTTTCTACTGGTGGTACGTGATCAGCCGTGCCTACGTCGAGCTACTACCACACTCTCGGCTCAATTACCGTGTGACATCGGATACTCCAACATGGCACGGCGACTGCTAGATGCGAGAAGCCTTACGTCTGCATAGACAGGTTAGTGCCAGCAGCCGCGGTAATTCCGATGACCTGCTTACCATGTTATAGCACCTCGTTTAGCAGCATGACGCAACTCGAGAGTCGCATTTGCGTAAACGGATACCATAGTGAAGTGCACATTCACGAAGTCCCTACCATAGAGCGGAATGTTCTAGAAGCCCGTTCGCAACACCTTAGGCCTATGGTTGAATTCACCGGTGTGGTCTCTGCACA

>ISG02

ATCACCTTCAAACTGGTCCAAGGTACACTAGGACGACGCGTCATTTGGGATTGGTTACGAAGGATGCCCTCACACCCATCCTACGGGAGGCAGCAGCGCCTTATACGGGGTTTCTACTGGTGGTTTCTCGGTTCATGGATCCACCTGTTACTTGCGGCTTGCCCACAAACGGTCCCTAGCGTTAGCCTTTGAAGAGAATAGATCACCTCCTATTATGGAATGAGCCGATCCTATGCTCATAGCCTGCACTCTGGTGCCAGCAGCCGCGGTAATCATGAAGGGTAATACGGGAAGACTAGTGACGGGTAAACTCCTTAGGACACAACTCACGTCAACCGCCGTTCAGATAGATAGGCCCTGTCGTCATACGTTCGCAAATGTTCCTTGGTACCCGGGTCATCATAGTCTGTTTGGGTAAACTGAACCGGCCAATCTCCTGAACAGACAAATCCGCCTCGCATATCT

>ISG03

CAGTGAAAGGGATGTGCTTGATACCGTGGGTATACCTCCGTGGTAATAAGCACCGTAATAAGTGAGTCCCGCGGGCTTACCTACGGGAGGCAGCAGTTGTGCTGCAGGGGTTTCTACTGGTGGTGTATAGCTACTATAGCGTAGGGATCGATATCAGCTATACCTAGATGAGAGCCCATTTCCGCTCGATATACCTAGGGACACGTAGATGTACCCTCTAGCAAGAGTTTGCCACAGATACGTACCGCTCATAGTGCCAGCAGCCGCGGTAAACGCGAACCGAAGCGCAGTAGAAGTACTCCGTATCCTACCTCGGTCGTGGTTTAGGCTATCGACATCTTGCATGGGCTTCCCTAGTGAACTCTACCCTTGTAACCTACACTGGTGTAAGACATCAGACAACGAACGCTAACTATCCTGCAACACTCCGGAAGAGATGAAGTCGCCGACGCGATACGAAACTTC

>ISG04

TCTCGCATGCATTCCGGCTCGGTAGTTACGGGTCGCGCATAAGAGTCGTACCGAAGCATTCCGTTATCCTAGGAACAAACCTACGGGAGGCAGCAGGGAAGTAGAGGGGGTTTCTACTGGTGGTTTACCGGACGACCGATGCATAGAACCGCTTATTCGCGGTACAAGTTGTGATGACGGAAACCTACGTCGAACGAAAGCGGCTAGCCCTTGGAACGCACTCTCGGATGTACCACACCAGACATGCGCCGTTGTGCCAGCAGCCGCGGTAAATGGATTATCAGGTTAGCCCAACAGGTTCGGTACATGTCGCTAGCTTGACTGGCCTAGGATCTCGAGATATCGGTGCACATCGTACCGAGATCACGCGAAATACTGCACTTACAGTAGCCATAGTCAGCATTAACGGGCGCATAAACTTACCGAGGAACCTTAAACGACACTGCGCTAAACGCTAGTGCGTCA

>*Methylomagnum ishizawai* strain RS11D-Pr

TCCGACTGGAAGGACCGCCGCCTGTGGGTCACCGTGACCCCGATCGTGCTGGTGACCTTCCCGGCCGCCGCGCAGGTGTTCCTGTGGGAACGTTTCCGCCAGCCGTGGGCCGCGACCGTGTGCGTGTTGGCGCTGCTGTTCGGTGAATGGGTCAACCGCTACTTCAACTTCTGGGGCTGGACCTACTTCCCCGTGAACTTCGTGTTCCCGGCCATCCTGGTCCCGGGCGCGATCATCCTCGACGTCTGCCTGATGCTGTCCGGCAGCTACCTGTTCACCGCCATCATCGGTGGCCTGGCCTGGGGTTTGATCTTCTACCCCGGCAACTGGCCGATCATCGCCCCGATCCACGTGCCGGTTGAATACAACGGCATGCTGATGTCCATCGCCGACATCCAGGGCTACAACTATGTGCGTACCGGCACCCCCGAGTACATCCGCATGGTCGAGAAGGGCACCCTGCGT

*amoA* gene amplicon sequence (*amoA* 1F/2R) (5**'**-3**'**)

>ISG01

ACGTGATCAGCCGTGCCTACGTCGAGCTACTACCACACTCTCGGCTCAATTACCGTGTGACATCGGATACTCCAACATGGCACGGCGACTGCTAGATGCGAGAAGCCTTACGTCTGCATAGACAGGTTAGTGCCAGCAGCCGCGGTAATTCCGATGACCTGCTTACCATGTTATAGCACCTCGTTTAGCAGCATGACGCAACTCGAGAGTCGCATTTGCGTAAACGGATACCATAGTGAAGTGCACATTCACGAAGTCCCTACCATAGAGCGGAATGTTCTAGAAGCCCGTTCGCAACACCTTAGGCCTATGGTTGAATTCACCGGTGTGGTCTCTGCACAACCTTCGGTAAGGATGTAATCCATGGCTAGGCGTTCTTCTCAGCGTTCTGTTCCATGTGGATTAGATACCCTGGTAGTCATCTACGTCCTAGGCCACGCCCTCCATAGATC

>ISG02

TTCTCGGTTCATGGATCCACCTGTTACTTGCGGCTTGCCCACAAACGGTCCCTAGCGTTAGCCTTTGAAGAGAATAGATCACCTCCTATTATGGAATGAGCCGATCCTATGCTCATAGCCTGCACTCTGGTGCCAGCAGCCGCGGTAATCATGAAGGGTAATACGGGAAGACTAGTGACGGGTAAACTCCTTAGGACACAACTCACGTCAACCGCCGTTCAGATAGATAGGCCCTGTCGTCATACGTTCGCAAATGTTCCTTGGTACCCGGGTCATCATAGTCTGTTTGGGTAAACTGAACCGGCCAATCTCCTGAACAGACAAATCCGCCTCGCATATCTACCTTCGGTAAGGATGTTCGAATACCTCTAGCGTTCTTCTCAGCGTTCGTCCGCTTCAGGATTAGATACCCTGGTAGTCAGTCTGTCCAAGTAAGCCATAGGCTGGAGGCC

>ISG03

GTATAGCTACTATAGCGTAGGGATCGATATCAGCTATACCTAGATGAGAGCCCATTTCCGCTCGATATACCTAGGGACACGTAGATGTACCCTCTAGCAAGAGTTTGCCACAGATACGTACCGCTCATAGTGCCAGCAGCCGCGGTAAACGCGAACCGAAGCGCAGTAGAAGTACTCCGTATCCTACCTCGGTCGTGGTTTAGGCTATCGACATCTTGCATGGGCTTCCCTAGTGAACTCTACCCTTGTAACCTACACTGGTGTAAGACATCAGACAACGAACGCTAACTATCCTGCAACACTCCGGAAGAGATGAAGTCGCCGACGCGATACGAAACTTCACCTTCGGTAAGGATGTTTACGCTAGGACTGCGTTCTTCTCAGCGTTCGAGCATCTACGGATTAGATACCCTGGTAGTCCCATTGCTCGATGTTAAGGGACCAAATGAGCC

>ISG04

TTACCGGACGACCGATGCATAGAACCGCTTATTCGCGGTACAAGTTGTGATGACGGAAACCTACGTCGAACGAAAGCGGCTAGCCCTTGGAACGCACTCTCGGATGTACCACACCAGACATGCGCCGTTGTGCCAGCAGCCGCGGTAAATGGATTATCAGGTTAGCCCAACAGGTTCGGTACATGTCGCTAGCTTGACTGGCCTAGGATCTCGAGATATCGGTGCACATCGTACCGAGATCACGCGAAATACTGCACTTACAGTAGCCATAGTCAGCATTAACGGGCGCATAAACTTACCGAGGAACCTTAAACGACACTGCGCTAAACGCTAGTGCGTCAACCTTCGGTAAGGATGTTCCGGCTTCATTCGCGTTCTTCTCAGCGTTCGTATGTCCGAGGATTAGATACCCTGGTAGTCTCTGTAGAGACTTCCCGACAAAGATTCGGCTG

>*Nitrosomonas europaea* ATCC 19718

CACACTACCCCATCAACTTCGTAACACCGGGCATTATGCTTCCGGGTGCGCTGATGCTGGACTTCACGCTGTATCTGACACGCAACTGGCTGGTGACGGCTCTGGTTGGAGGTGGATTCTTCGGTCTGCTGTTCTATCCGGGTAACTGGCCGATTTTTGGACCAACCCATTTGCCAATCGTTGTAGAAGGCACATTGCTGTCGATGGCTGATTACATGGGACATCTGTATGTTCGTACAGGTACACCCGAGTATGTTCGTCATATTGAGCAAGGTTCACTGCGTACCTTTGGTGGTCATACCACAGTTATTGCAGCATTCTTCTCTGCGTTCGTATCAATGTTGATGTTCACCGTATGGTGGTATCTTGGAAAAGTTTACTGTACAGCCTTTTTCTACGTTAAAGGTAAAAGAGGTCGTATCGTACATCGCAATGATGTTACCGCATTCGGT

**Table S3:** Internal standard gene sequences, including capture regions of *pmoA*, *amoA*, and 16S rRNA genes.

| **Internal Standard Gene** | **Length (bp)** | **GC (%)** | **Sequence (5'-3')** |
| --- | --- | --- | --- |
| ISG01 | 615 | 52.8 | GGCGACTGGGACTTCTGGGCACGGAAATAAGACGTGCCGACAAATTCCTCTTATGACCAAAGTGGGCGTCCATGGCTTAGACTCGTGTGGCTCGAACCCTACGGGAGGCAGCAGCGAAGTCTTGGGGGTTTCTACTGGTGGTACGTGATCAGCCGTGCCTACGTCGAGCTACTACCACACTCTCGGCTCAATTACCGTGTGACATCGGATACTCCAACATGGCACGGCGACTGCTAGATGCGAGAAGCCTTACGTCTGCATAGACAGGTTAGTGCCAGCAGCCGCGGTAATTCCGATGACCTGCTTACCATGTTATAGCACCTCGTTTAGCAGCATGACGCAACTCGAGAGTCGCATTTGCGTAAACGGATACCATAGTGAAGTGCACATTCACGAAGTCCCTACCATAGAGCGGAATGTTCTAGAAGCCCGTTCGCAACACCTTAGGCCTATGGTTGAATTCACCGGTGTGGTCTCTGCACAACCTTCGGTAAGGACGTAATCCATGGCTAGGCGTTCTTCTCAGCGTTCTGTTCCATGTGGATTAGATACCCTGGTAGTCATCTACGTCCTAGGCCACGCCCTCCATAGATCGAAGAAGGCTTTCCAGAGGGG |
| ISG02 | 615 | 52.0 | GGCGACTGGGACTTCTGGATCACCTTCAAACTGGTCCAAGGTACACTAGGACGACGCGTCATTTGGGATTGGTTACGAAGGATGCCCTCACACCCATCCTACGGGAGGCAGCAGCGCCTTATACGGGGTTTCTACTGGTGGTTTCTCGGTTCATGGATCCACCTGTTACTTGCGGCTTGCCCACAAACGGTCCCTAGCGTTAGCCTTTGAAGAGAATAGATCACCTCCTATTATGGAATGAGCCGATCCTATGCTCATAGCCTGCACTCTGGTGCCAGCAGCCGCGGTAATCATGAAGGGTAATACGGGAAGACTAGTGACGGGTAAACTCCTTAGGACACAACTCACGTCAACCGCCGTTCAGATAGATAGGCCCTGTCGTCATACGTTCGCAAATGTTCCTTGGTACCCGGGTCATCATAGTCTGTTTGGGTAAACTGAACCGGCCAATCTCCTGAACAGACAAATCCGCCTCGCATATCTACCTTCGGTAAGGACGTTCGAATACCTCTAGCGTTCTTCTCAGCGTTCGTCCGCTTCAGGATTAGATACCCTGGTAGTCAGTCTGTCCAAGTAAGCCATAGGCTGGAGGCCGAAGAAGGCTTTCCAGAGGGG |
| ISG03 | 615 | 51.5 | GGCGACTGGGACTTCTGGCAGTGAAAGGGATGTGCTTGATACCGTGGGTATACCTCCGTGGTAATAAGCACCGTAATAAGTGAGTCCCGCGGGCTTACCTACGGGAGGCAGCAGTTGTGCTGCAGGGGTTTCTACTGGTGGTGTATAGCTACTATAGCGTAGGGATCGATATCAGCTATACCTAGATGAGAGCCCATTTCCGCTCGATATACCTAGGGACACGTAGATGTACCCTCTAGCAAGAGTTTGCCACAGATACGTACCGCTCATAGTGCCAGCAGCCGCGGTAAACGCGAACCGAAGCGCAGTAGAAGTACTCCGTATCCTACCTCGGTCGTGGTTTAGGCTATCGACATCTTGCATGGGCTTCCCTAGTGAACTCTACCCTTGTAACCTACACTGGTGTAAGACATCAGACAACGAACGCTAACTATCCTGCAACACTCCGGAAGAGATGAAGTCGCCGACGCGATACGAAACTTCACCTTCGGTAAGGACGTTTACGCTAGGACTGCGTTCTTCTCAGCGTTCGAGCATCTACGGATTAGATACCCTGGTAGTCCCATTGCTCGATGTTAAGGGACCAAATGAGCCGAAGAAGGCTTTCCAGAGGGG |
| ISG04 | 615 | 52.8 | GGCGACTGGGACTTCTGGTCTCGCATGCATTCCGGCTCGGTAGTTACGGGTCGCGCATAAGAGTCGTACCGAAGCATTCCGTTATCCTAGGAACAAACCTACGGGAGGCAGCAGGGAAGTAGAGGGGGTTTCTACTGGTGGTTTACCGGACGACCGATGCATAGAACCGCTTATTCGCGGTACAAGTTGTGATGACGGAAACCTACGTCGAACGAAAGCGGCTAGCCCTTGGAACGCACTCTCGGATGTACCACACCAGACATGCGCCGTTGTGCCAGCAGCCGCGGTAAATGGATTATCAGGTTAGCCCAACAGGTTCGGTACATGTCGCTAGCTTGACTGGCCTAGGATCTCGAGATATCGGTGCACATCGTACCGAGATCACGCGAAATACTGCACTTACAGTAGCCATAGTCAGCATTAACGGGCGCATAAACTTACCGAGGAACCTTAAACGACACTGCGCTAAACGCTAGTGCGTCAACCTTCGGTAAGGACGTTCCGGCTTCATTCGCGTTCTTCTCAGCGTTCGTATGTCCGAGGATTAGATACCCTGGTAGTCTCTGTAGAGACTTCCCGACAAAGATTCGGCTGGAAGAAGGCTTTCCAGAGGGG |

**Table S4:** Primer sets for amplification of functional internal standard sequences.

| Gene target | Primer name | Primer sequence (5'-3') | Product length (bp) | Reference |
| --- | --- | --- | --- | --- |
| Synthetic internal standard sequences | ISG_F_ampli | GGCGACTGGGACTTCTGG | 615 | Modified Holmes *et al.*, 1995 |
|  | ISG_R_ampli | CCCCTCTGGAAAGCCTTCTTC |  | Modified Rotthauwe *et al.*, 1997 |
| Thermocycling protocol |  |  |  |  |
| Initial heat activation, 95°C 15min; Amplification for 30 cycles, 94°C 30s, 55°C 30s, 72°C 1min; Final extension, 72°C 10min | | | | |

**Table S5:** Primer lists and thermocycling protocols for qPCR standard curve preparation.

| Gene target | Primer name | Primer sequence (5'-3') | Product length (bp) | Reference |
| --- | --- | --- | --- | --- |
| *Methylomagnum ishizawai*  strain RS11D-Pr *pmoA*^a^ | *pmoA* 189f | GGNGACTGGGACTTCTGG | 531 | Holmes *et al.*, 1995 |
|  | *pmoA* 682r | GAASGCNGAGAAGAASGC |  |  |
| *Nitrosomonas europaea*  ATCC 19718 *amoA*^b^ | *amoA* 1F | GGGGTTTCTACTGGTGGT | 491 | Rotthauwe *et al.*, 1997 |
|  | *amoA* 2R | CCCCTCKGSAAAGCCTTCTTC |  |  |
| *Escherichia coli* NBRC 3301  16S rRNA V1-9 region^c^ | 27F | AGAGTTTGATCMTGGCTCAG | 1506 | Weisburg *et al.*, 1991 |
|  | 1492R | TACGGYTACCTTGTTACGACTT |  |  |
| Illumina adapter sequence |  | | | |
| Forward sequence, 5’-TCGTCGGCAGCGTCAGATGTGTATAAGAGAAG-3’ | | | | |
| Reverse sequence, 5’-GTCTCGTGGGCTCGGAGATGTGTATAAGAGACAG-3’ | | | | |
| Thermocycling protocol |  |  |  |  |
| a. Initial heat activation, 95°C 15min; Amplification for 35 cycles, 94°C 30s, 56°C 1min, 72°C 1min; Final extension, 72°C 10min | | | | |
| b. Initial heat activation, 95°C 15min; Amplification for 30 cycles, 94°C 30s, 55°C 40s, 72°C 1min; Final extension, 72°C 10min | | | | |
| c. Initial heat activation, 95°C 15min; Amplification for 33 cycles, 94°C 30s, 55°C 30s, 72°C 2min; Final extension, 72°C 10min | | | | |

**Reference**

Holmes, A.J., Costello, A., Lidstrom, M.E., and Murrell, J.C. (1995) Evidence that participate methane monooxygenase and ammonia monooxygenase may be evolutionarily related. *FEMS Microbiol Lett* **132**: 203–208.

Ramakodi, M.P. (2021) A comprehensive evaluation of single-end sequencing data analyses for environmental microbiome research. *Arch Microbiol* **203**: 6295–6302.

Rotthauwe, J.H., Witzel, K.P., and Liesack, W. (1997) The ammonia monooxygenase structural gene amoa as a functional marker: Molecular fine-scale analysis of natural ammonia-oxidizing populations. *Appl Environ Microbiol* **63**: 4704–4712.

Weisburg, W.G., Barns, S.M., Pelletier, D.A., and Lane, D.J. (1991) 16S ribosomal DNA amplification for phylogenetic study. *J Bacteriol* **173**: 697–703.
